# Interactions between physiology and behaviour provide insights into the ecological role of venom in Australian funnel-web spiders: Interspecies comparison

**DOI:** 10.1371/journal.pone.0285866

**Published:** 2023-05-22

**Authors:** Linda Hernández Duran, David Thomas Wilson, Mohamed Salih, Tasmin Lee Rymer

**Affiliations:** 1 College of Science and Engineering, James Cook University, Cairns, Australia; 2 Centre for Tropical Environmental and Sustainability Sciences, James Cook University, Cairns, Australia; 3 Australian Institute for Tropical Health and Medicine, Centre for Molecular Therapeutics, James Cook University, Cairns, Australia; 4 Department of Obstetrics and Gynaecology, Monash University, Clayton, Victoria, Australia; Instituto Butantan, BRAZIL

## Abstract

Australian funnel-web spiders are iconic species, characterized as being the most venomous spiders in the world. They are also valued for the therapeutics and natural bioinsecticides potentially hidden in their venom molecules. Although numerous biochemical and molecular structural approaches have tried to determine the factors driving venom complexity, these approaches have not considered behaviour, physiology and environmental conditions collectively, which can play a role in the evolution, complexity, and function of venom components in funnel-webs. This study used a novel interdisciplinary approach to understand the relationships between different behaviours (assessed in different ecological contexts) and morphophysiological variables (body condition, heart rate) that may affect venom composition in four species of Australian funnel-web spiders. We tested defensiveness, huddling behaviour, frequency of climbing, and activity for all species in three ecological contexts: i) predation using both indirect (puff of air) and direct (prodding) stimuli; ii) conspecific tolerance; and iii) exploration of a new territory. We also assessed morphophysiological variables and venom composition of all species. For *Hadronyche valida*, the expression of some venom components was associated with heart rate and defensiveness during the predation context. However, we did not find any associations between behavioural traits and morphophysiological variables in the other species, suggesting that particular associations may be species-specific. When we assessed differences between species, we found that the species separated out based on the venom profiles, while activity and heart rate are likely more affected by individual responses and microhabitat conditions. This study demonstrates how behavioural and morphophysiological traits are correlated with venom composition and contributes to a broader understanding of the function and evolution of venoms in funnel-web spiders.

## Introduction

Behaviour is known as the “evolutionary pacemaker” because animals are able to either change or maintain particular behaviours when exposed to different environmental conditions [1,2]. Individual behavioural phenotypes (i.e., aggressive, docile, active) have considerable impact at larger scales, affecting population dynamics, colonisation of new habitats, trophic interactions, distribution and extinction rates [3–5]. Therefore, changes in the environment and behaviour can mediate the evolution of morphology, life history traits [3,4], and physiology, such as venom.

Venom is used across multiple ecological contexts that extend beyond predator-prey interactions [2]. Some animals can use venom for communication [6], predation [1], defence [1,7], mating [8], intraspecific competition [9,10], and social behaviour [11]. The use of venoms can involve different toxins, and various combinations of toxins can result in the ability to perform multiple functions, making venom a unique adaptive biological trait [1,2]. In venomous animals, behaviour plays a critical role because the use of venoms is mediated by behavioural responses [1]. Venom function and molecular variation are thus constrained by behavioural responses, in which costs of production, such as high metabolic rates [12,13], replenishment, deployment and availability of venom, are moderated by selection [1,14].

Multiple extrinsic (environment, diet, density) and intrinsic (genes, physiology) factors also directly or indirectly affect behaviour, which can in turn affect the use, function and composition of venom [2]. To date, seasonality [15], sexual dimorphism [16–19], age [20], ontogeny [21,22], geographical origin [23], genetics [24], and environmental conditions [25] have been studied independently to understand their effects on the variation in venom composition. However, intrinsic and extrinsic factors interact, affecting physiological and behavioural processes that can vary at a species-specific level [26]. Understanding the associations between behaviour, physiology and environment is important because the ecological function of venom depends on how an animal responds to a particular cue (threat level [27]), and the strategies developed by them to deploy the venom [1]. For example, physiological traits like body condition (i.e., nutritional state) can affect the performance of animals in a particular context. Individuals in better body condition may take more risks (be more bold) or show more defensive displays [28], which can then balance the metabolic costs [29] of using and producing venom [30,31].

The costs associated with venom use can also vary depending on ecological context and level of threat [1,2,27]. Defensive and exploratory behaviours usually consume energy, whereas foraging and defending food supplies compensate for costs associated with energy expenditure [32]. In the case of venomous animals, venom production is energetically expensive [1] and the nutritional state of an individual can increase or decrease the costs of venom expenditure [1,13]. Venomous animals can compensate for the energetic costs (metabolic rate or observed when heart rate frequency increases [33,34]) of venom expenditure using multiple strategies, such as adjusting bites [27], using behavioural displays [35,36] or controlling the amount of venom expelled [1,13,37].

Australian funnel-web spiders (family Atracidae, consisting of the genera *Atrax*, *Hadronyche* and *Illawara*) are iconic species endemic to the southern and eastern regions of the continent [38]. Together with the sister lineages Actinopodidae (genera *Actinopus*, *Missulena* and *Plesiolena*) and Macrothelidae (genus *Macrothele*) comprise several medically important species, and collectively make up “the venom clade” [39]. Funnel-web venoms have diverse pharmacological activities [40], and are the most complex venoms in the natural world (comprising thousands of toxin peptides) [40]. Funnel-web spiders show inter- and intra-individual differences in venom composition, as well as interspecific differences between closely related species with overlapping distributions [41]. Numerous biochemical and molecular structural approaches have explored the factors affecting these complex venoms [19]. However, aspects related to the ecological function of venom, physiological traits, and their interactions with behaviour, have been overlooked. In this study, we used a synergistic approach to assess whether behavioural and morphophysiological traits measured over three ecological contexts (predation, conspecific tolerance, and exploration of a new territory) affect venom composition in four species of Australian funnel-web spiders: *Hadronyche valida*, *H*. *infensa*, *H*. *cerberea* and *Atrax robustus*. Each species varies in habitat and venom composition [26], thus representing an ideal model for understanding how different factors interact to affect venom diversity within and between species. Within species we expected that the ecological context (e.g. predator defence) would affect relationships with venom components and morphophysiological traits, where defensive and more active spiders would have higher heart rates and show expression of specific venom molecules because displaying defensive behaviour and expelling venom are costly traits involving energy expenditure. Furthermore, while species differences in venom composition are well known [41,42], species differences in behaviour and morphophysiological traits, and their interactions with venom have been overlooked. We expected *H*. *infensa* and *H*. *valida* to be more similar in their behavioural and physiological responses because they are sympatric, occupy similar habitats and are both burrow dwellers [38]. In contrast, we expected that *H*. *cerberea* would be less similar to the other *Hadronyche* species and *A*. *robustus* because it is the only tree-dweller [38]. This study allowed us to identify physiological changes (heart rate and venom components) associated with a particular ecological context and behaviour, which is not only important for understanding the mechanisms underlying the evolution of venoms and toxins but is also important for understanding how animals use their biological weapons to respond to different environmental conditions.

## Methods

### Study species

For this study, 75 spiders from four different Australian funnel-web spider species were used. Adult females of *H*. *valida* (n = 23; Currumbin Valley and Mount Tamborine) were purchased from Thargomindah Man Productions (Varsity Lakes, QLD, Australia) in 2019. Adult females of *H*. *infensa* (n = 16) were collected manually in Toowoomba and Ravensbourne in 2019 (collection permit SA 2016/08/55). *H*. *cerberea* (n = 9 adult females; n = 9 juveniles) were collected across three *Eucalyptus regnans* in Gosford, New South Wales in 2019. *A*. *robustus* (n = 13 adult females; n = 5 juveniles) were collected from the Gosford/Central Coast region, New South Wales. Ccollection of *H*. *cerberea* and *A*. *robustus* were carried out in cooperation with the Australian Reptile Park. We collected any spiders we could find, regardless of size or stage, which led to unbalanced sample sizes. The collection of these animals is particularly difficult as spiders live in burrows [38], which made it difficult to know whether the spider being collected was an adult or juvenile prior to collection. Adult females were identified by sclerotisation of the spermathecae after moulting and the opening of the epigastric furrow [41,43]. The spiders were transported alive in plastic containers with damp cotton wool to the laboratory of the Australian Institute of Tropical Health and Medicine (AITHM), James Cook University Nguma-bada (Cairns) campus, Queensland, Australia.

Spiders were kept individually in 5 L plastic containers in a climate-controlled room (temperature: 20 ± 2°C; relative humidity: 60%) on a reverse light:dark cycle (12L:12D). The spiders were acclimated for one month before commencing behavioural assays (see below). Each spider received one house cricket, *Acheta domestica*, once a week. Two weeks before starting all behavioural tests, food was withheld to allow venom regeneration prior to collection. All behaviours were video recorded using a Sony Handycam under red light between 6 am and 12 pm, and were later analysed using the software BORIS version 7.8.2 [44].

### Behavioural assays

Multiple behavioural traits (defence, huddling, defence towards conspecifics, and activity) were measured across three ecological contexts namely predation, conspecific tolerance, and exploration of new territory for each species. The methods are described and discussed in [41,45], which we summarise here briefly. The number of fang movements produced in response to a prod stimulus (using blunt tweezers to touch the first pair of legs) was quantified as a measure of defence. As a measurement of risk-taking behaviour (antipredator behaviour), we used huddling behaviour in response to three rapid puffs of air applied on the prosoma using a camera air blower. Defence towards conspecifics was measured using the number of climbs on a barrier when the spiders were exposed to a conspecific (spiders divided by a mesh barrier in a novel environment to avoid direct agonistic encounters). Finally, as a measure of exploration of a new territory, we used the time the spider spent actively moving around a new arena.

The average value of each behavioural trait measured across different ecological contexts and over time (three repetitions with one month between each repetition) were obtained. We previously assessed repeatability and behavioural flexibility in all four species [46], so do not discuss this here. The behaviours from both adults and juveniles for *H*. *cerberea* and *A*. *robustus* were analysed together as a longitudinal analysis found no effect of stage differences over time for any of the behavioural traits, heart rate and body condition (see statistical analyses; S1 Table).

### Morphological and physiological measurements

For each species, one day after we finalised each behavioural assay, we measured body size (cephalothorax width) and body mass (weight) of each spider. We calculated the mean of body size and mass from all repetitions. We tested the normality of each variable using the MVN package [47]. Then, we calculated body condition using the residual index, in which body mass is regressed against body size [48]. We used a linear model (LM) with normal distribution to obtain the residuals.

#### Heart rate

Individual heartbeats have been used as an accurate measure of heart rate in spiders [33]. Heart rate can be used as a standard measurement of metabolic rate in spiders [33]. We measured the heart rate of each individual spider for each species in a resting position [49] one day after finishing all the behavioural tests and feeding the spiders (to reduce the effects of any stressful conditions). The heart rate was measured using a non-invasive laser heart rate monitor (S1 Appendix) [50,51]. Spiders were gently extracted from their burrows and left in a resting position for one hour. We placed the heart rate monitor sensor close and pointing at the abdomen of the spider, avoiding direct physical contact. The sensor fires harmless infrared wavelengths towards the spider and reflection from the heart returns the number of beats within a 15 s monitoring period. We measured the heart rate at least 4 times within each measurement period to ensure that the monitor was functioning correctly. Then, we calculated the overall mean from all repetitions for each spider.

### Venom collection and analysis

For each species, during the defence test, we collected the venom expelled on the tips of the fangs of each spider using a 200μL Gilson P200 pipette with polypropylene micropipette [36,41]. Each spider was aggravated for 4 min and venom was collected over a 10 min period. After collecting the venom, it was placed in a 1.5 mL microcentrifuge tube with 40 μL of Milli-Q water [41]. We repeated this procedure three more times, one month apart, for a total of three venom samples per individual. Venom samples from each individual were analysed using liquid chromatography/electrospray ionisation mass spectrometry (LC/ESI-MS) to generate a venom profile, following the protocol of [41].

#### Matrices

*Within species*. To analyse the relationship between venom components and both behavioural and morphophysiological variables (heart rate and body condition), we built three matrices: one matrix included the mean of the three repetitions of each behaviour (defence, huddling, defence towards conspecifics, and activity), heart rate and body condition. The second matrix was built from the total of all venom components present across all three repetitions. The low molecular weights obtained from singly charged ions were monoisotopic masses, while those obtained from reconstructed multi-charged ion series were average masses.

The third matrix consisted of a reduced matrix of venom components, which was obtained after performing a Spearman rank correlation to determine whether there was redundancy in the data. We first performed a multivariate analysis using the complete venom matrix, behavioural and morphophysiological variables. We then repeated this analysis using the reduced matrix of venom components, behavioural and morphophysiological variables. We included both complete and reduced venom matrices to determine if the association between traits dramatically changed after reducing the redundancies between venom components. In addition, keeping both matrices allowed us to visualise the association between particular venom molecules with behavioural and morphophysiological traits that might not be present if only one matrix was used.

#### Between species comparison

To assess interspecific relationships based on venom profiles, behaviours and morphophysiological traits, we followed the same protocol described above, firstly building a matrix that included all behaviours, heart rate and body condition from all individuals from all species, then building a matrix that included venom profiles from all individuals from all species. We then reduced the redundancy in venom components (by performing a Spearman rank correlation, cut-off 0.70) and built a reduced venom matrix.

### Statistical analyses

All statistical analyses were conducted using R (version 4.1.0). For both adults and juveniles of *H*. *cerberea* and *A*. *robustus*, we tested for stage effects on the magnitude of individual behavioural traits (defence, huddling, defence towards conspecifics and activity), heart rate and body condition in each ecological context, and over time, using rank-based non-parametric analyses for longitudinal data (S1 Table) following [36]. These analyses offer a robust framework for non-continuous variables, small sample sizes and skewed data [52]. The design used was F1-LD-F1 in the nparLD package [52]. We included the random effect of individual identity as a subject in this model. We did not observe differences between adults and juveniles for either *H*. *cerberea* or *A*. *robustus* for behavioural traits, heart rate and body condition (S1 Table). Therefore, we did not separate juvenile and adult data for each species when the multivariate analyses were performed.

#### Within species analysis

To determine the relationship between venom components with behavioural (defence, huddling, climbing and activity) and morphophysiological (heart rate and body condition) variables in each species, we used a multivariate method canonical correspondence analysis (CCA) using the package vegan [53]. The collinearity of explanatory variables (behaviours and morphophysiological variables centered) was measured using the variance inflation factor (VIF) [54]. We first ran the CCA using the venom matrix with all venom components, where the Chi-square distance was used by default in the package. Then, we re-ran the analysis using the Bray-Curtis distance, which is more suitable for abundance data [54], to determine which model had the better fit (S2 Table). We repeated this procedure for the reduced venom matrix (S3 Table).

Furthermore, to visualise the correlation structure between venom profiles with behavioural and morphophysiological variables, we used Regularised Canonical Correlation Analysis (rCCA) from the mixOmics package [55]. This package uses omics data and it is particularly suitable when the number of samples (N) is lower than the number of variables provided in two matrices (X and Y) [55]. This method achieves dimension reduction in each data set while maximising similar information within species matrices. The regularisation parameter used in rCCA was cross-validation [55]. The rCCA was performed for the complete matrix of venom components, and then the same analysis was conducted using the reduced matrix of venom components. Finally, using the rCCA results, we built network and cluster image map (CIM) plots to evaluate the correlation structure between venom components with behavioural and morphophysiological variables [55].

#### Between species comparison

To assess how individuals of each species grouped according to venom profiles, we performed nonmetric multidimensional scaling (NMDS) using the package vegan [53] after reducing the collinearity of venom components shared between species. We used Hellinger transformation and Bray-Curtis dissimilarity distance to separate groups based on the abundancies of venom components [53]. To determine the effect of behaviours and morphophysiological variables on the ordination plot NMDS of all venom profiles, we performed a permutation test using the envfit package [54].

### Ethical statement

Spiders were observed daily and monitored weekly. Experimental procedures did not have any negative effects on the animals. No spiders died during the experiment, and spiders readily resumed feeding following behavioural tests. As funnel-web spiders are not protected species in Australia, a scientific permit was not required. Collection of *H*. *infensa* from Ravensbourne National Park were collected under the Queensland Department of Environment and Science collection permit of Mr Rod Hobson (collection permit SA 2016/08/55). Our research was conducted within the framework of the Australian Code for the Care and Use of Animals for Scientific Purposes [56].

## Results

### Within species: Complete matrix for *H*. *valida*

The canonical correspondence analysis of the complete venom matrix (95 venom components, Chi-square distance) for *H*. *valida* showed that the model as a whole (with all the variables included) was significant (CCA, permutation test, F_6,16_ = 1.455, P = 0.047, Table 1). However, none of the individual canonical axes was significant (S2A Table). The amount of constrained variance explained in the venom matrix by the behavioural and morphophysiological variables was 27%. The variables that had the strongest effect on the venom matrix were heart rate (CCA, F_1,16_ = 2.156, P = 0.037; Table 1) and, marginally significant, climbing (CCA, F_1,16_ = 1.985, P = 0.059; Table 1). Similar results were obtained using the Bray-Curtis distance, where the whole model was significant (CCA, permutation test, F_6,16_ = 1.455, P = 0.044; Table 1), but none of the canonical axes were significant (S2D Table). Both heart rate (CCA, F_1,16_ = 2.156, P = 0.056; Table 1) and climbing (CCA, F_1,16_ = 1.985, P = 0.058; Table 1) had marginally significant effects on the venom matrix. No other variables had an effect (S2D and S2E Table).

**Table 1:** Results from ordination analysis. CCA of *H*. *valida* showing the relationship between venom components matrix (complete and reduced), behavioural and morphophysiological variables assessed across different ecological contexts using two different distances (Chi-square and Bray-Curtis).

Model:	Full venom matrix + Morphophysiological variables (Chi-square)	Full venom matrix + Morphophysiological variables (Bray-Curtis)	Reduced + Morphophysiological variables (Chi-square)	Reduced+ Morphophysiological variables (Bray-Curtis)
**Inertia**	0.779	0.779	0.881	0.881
Constrained	0.275	0.278	0.311	0.311
unconstrained	0.504	0.502	0.570	0.570
Accumulated CCA1	0.313	0.313	0.239	0.239
Accumulated CCA2	0.231	0.239	0.225	0.225
**ANOVA whole model**	**0.047**	**0.044**	**0.012**	**0.016**
**Behaviour/ morphophysiological variable (P.value)**				
Heart rate	**0.037**	0.056·	**0.031**	**0.040**
Climbing	0.059**·**	0.058·	0.096**·**	0.108
Defence	0.100	0.100	**0.013**	**0.024**
Body condition	0.190	0.172	0.096**·**	0.086·
Activity	0.286	0.268	0.097**·**	0.083·
Huddle	0.249	0.253	0.205	0.227
**R2 adjusted**	0.1088	0.1138	0.1129	0.111

#### Reduced matrix for *H*. *valida*

After performing a Spearman rank correlation, the venom matrix was reduced from 95 to 53 venom components. The CCA using the reduced matrix and the Chi-square distance improved the model fit (CCA, permutation test, F_6,16_ = 1.455, P = 0.012, Table 1). Again, none of the canonical axes were significant (S3A Table). The behaviour and morphophysiological variables explained 31% of variance in the venom matrix. The morphophysiological variable that had the strongest effect on the venom matrix was again heart rate (CCA, F_1,16_ = 1.893, P = 0.031; Table 1). However, this time, defence (CCA, F_1,16_ = 1.940, P = 0.013; Table 1), rather than climbing had a larger effect. Climbing (CCA, F_1,16_ = 1.567, P = 0.096; Table 1), body condition (CCA, F = 1.442, P = 0.098; Table 1), and activity (CCA, F_1,16_ = 1.446, P = 0.097; Table 1), while non-significant, were the next most important contributors.

**Fig 1:**
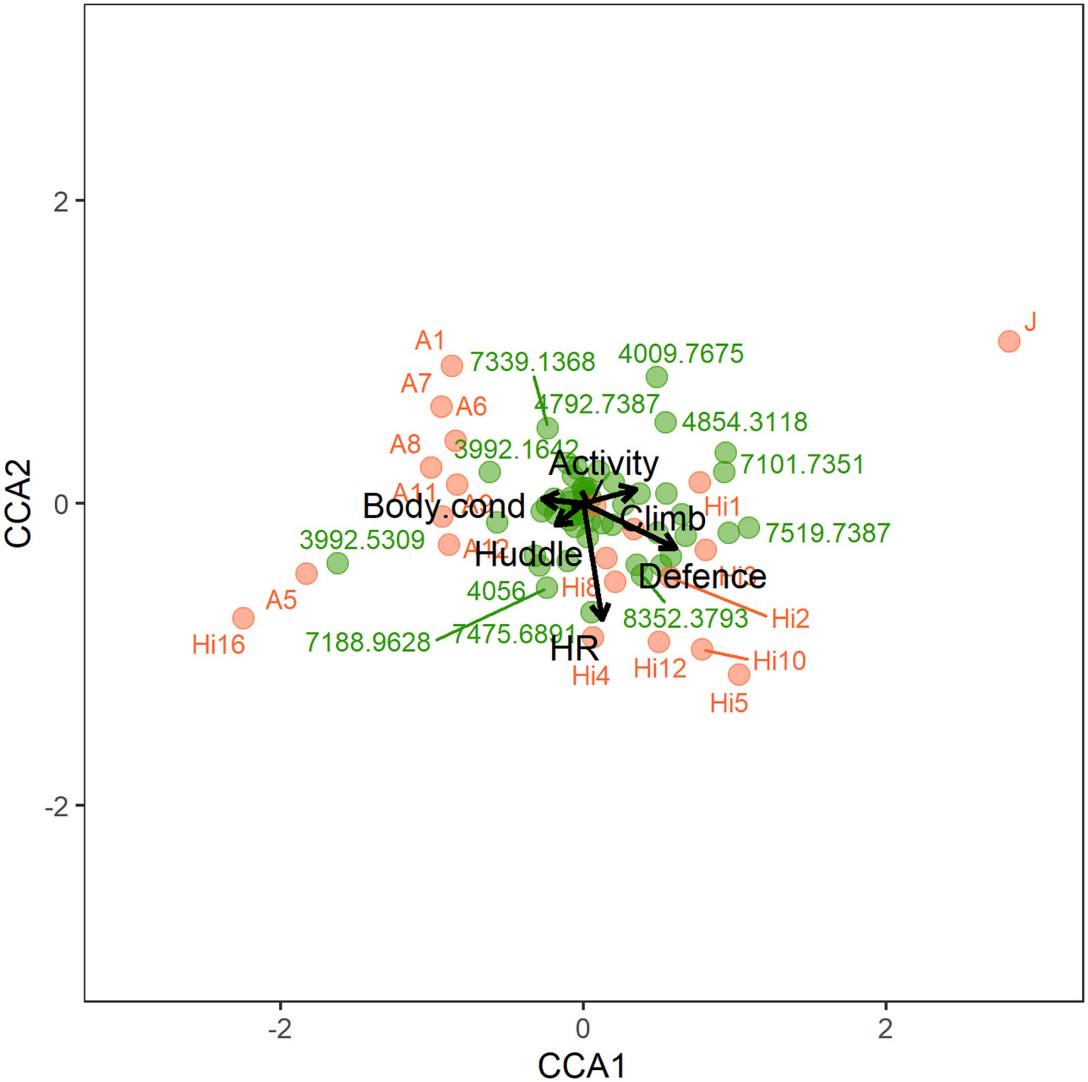
Canonical correspondence analysis (CCA) of the reduced venom profile matrix. Used the Bray-Curtis distance (green letters), with the matrix constrained by behavioural and morphophysiological (black letters) variables of the individuals (red letters) of *Hadronyche valida*.

The CCA results using the Bray-Curtis distance were similar to the model using the Chi-square distance (Table 1, Fig 1). The whole model was significant (CCA, permutation test, F_6,16_ = 1.455, P = 0.016, Table 1, Fig 1), but none of the individual canonical axes were significant (S3B Table). With regards to the effect of the behavioural and morphophysiological variables, heart rate (CCA, F_1,16_ = 1.893, P = 0.040; Table 1) and defence (CCA, F_1,16_ = 1.940, P = 0.024; Table 1) had significant effects on the venom matrix. Body condition (CCA, F_1,16_ = 1.442, P = 0.086; Table 1, Fig 1) and activity (CCA, F_1,16_ = 1.446, P = 0.083; Table 1), while non-significant, were the next most important contributors.

**Fig 2:**
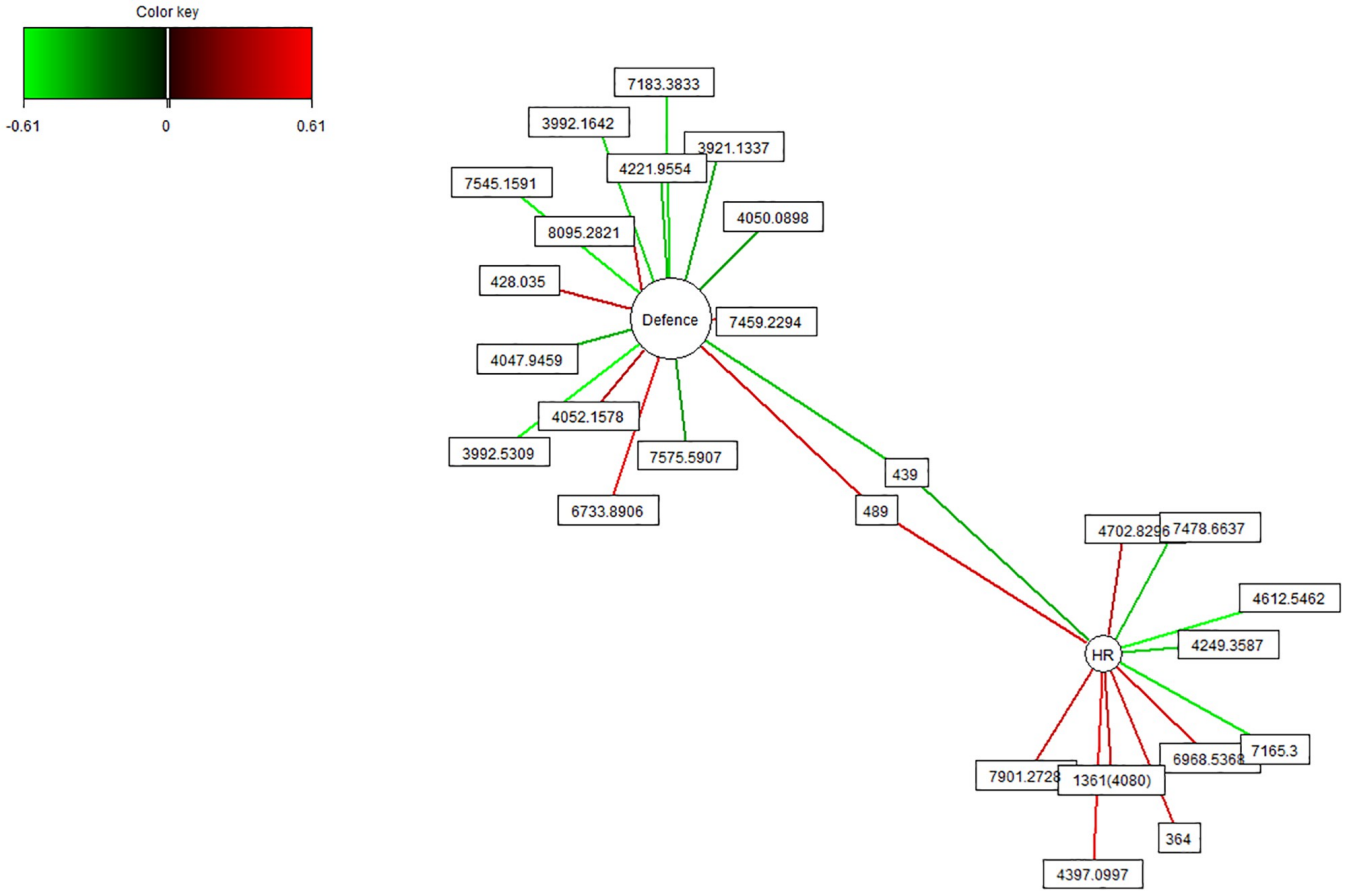
Network plot *Hadronyche valida* of a regularised canonical correlation analysis (rCCA). Showing the structure of the association of venom components (complete venom matrix) with behavioural and morphophysiological variables. The correlation cut-off shown is at 0.40. The colour of each line indicates the nature of the correlation (red: Positive association, green: Negative association) between venom components, defence, and heart rate.

The network plot showed that heart rate and defence had the strongest correlations with some venom molecules (Fig 2), which was supported by the rCCA analysis for both the complete and reduced venom matrices (S1 and S3 Figs). In particular, we found that two venom molecules were shared between heart rate and defence for the complete venom matrix (Fig 2). The venom molecule 439 Da was negatively associated, and the molecule 489 was positively associated with defence and heart rate (Fig 2). For the reduced venom matrix, the venom molecules 489 Da and 4397.099 Da showed a positive association with both heart rate and defence, while venom molecule 4612.546 Da showed a negative association with these two traits (S2 Fig). In addition, other specific venom molecules were associated either with defence or heart rate for both the complete venom matrix (Fig 2) and the reduced venom matrix (S2 Fig).

The CIM plots showed that both the complete and reduced venom matrices separated into two main clusters (S4A and S4B Fig). In the first cluster, defence and heart rate were grouped together, and showed various associations with specific venom molecules. In the second cluster, climbing, activity, body condition and huddle were clustered together. Both clusters were either positively or negatively associated with a particular venom component (S4A and S4B Fig).

#### Complete and reduced matrices for the other three species

The CCAs using the complete and reduced venom matrices with the Chi-square distance for *H*. *infensa*, *H*. *cerberea* and *A*. *robustus* individually were not significant (Table 2). Similarly, Bray-Curtis did not improve the model in any species (Table 2).

**Table 2:** Results from the ordination analysis including *H*. *infensa*, *H*. *cerberea* and *A*. *robustus*. CCA showing the relationship between the venom components matrix (complete and reduced) and behavioural and morphophysiological variables assessed across different ecological contexts using two different distances (Chi-square and Bray-Curtis).

*H*. *infensa*				
Model:	Complete venom matrix + Morphophysiological variables (Chi-square)	Reduced venom matrix + Morphophysiological variables (Bray-Curtis)	Complete venom matrix + Morphophysiological variables (Chi-square)	Reduced venom matrix + Morphophysiological variables (Bray-Curtis)
**Inertia**	0.693	0.693	0.689	0.689
**Constrained**	0.260	0.260	0.266	0.266
**Unconstrained**	0.432	0.432	0.423	0.423
**Accumulated CCA1**	0.284	0.284	0.289	0.289
**Accumulated CCA2**	0.190	0.190	0.288	0.288
**ANOVA (P.value)**	0.717	0.709	0.690	0.675
**F**	0.903	0.903	0.944	0.944
***H*. *cerberea***				
**Inertia**	0.585	0.585	0.585	0.772
**Constrained**	0.193	0.193	0.193	0.262
**Unconstrained**	0.392	0.392	0.392	0.509
**Accumulated CCA1**	0.285	0.285	0.285	0.305
**Accumulated CCA2**	0.262	0.262	0.262	0.206
**ANOVA (P.value)**	0.727	0.690	0.667	0.626
**F**	0.900	0.906	0.946	0.946
***A*. *robustus***				
**Inertia**	2.878	2.878	1.644	1.644
**Constrained**	0.989	0.989	0.498	0.498
**Unconstrained**	1.889	1.889	1.145	1.145
**Accumulated CCA1**	0.270	0.270	0.233	0.233
**Accumulated CCA2**	0.248	0.248	0.196	0.196
**ANOVA (P.value)**	0.588	0.588	0.998	0.998
**F**	0.960	0.960	0.798	0.798

### Between species

**Fig 3:**
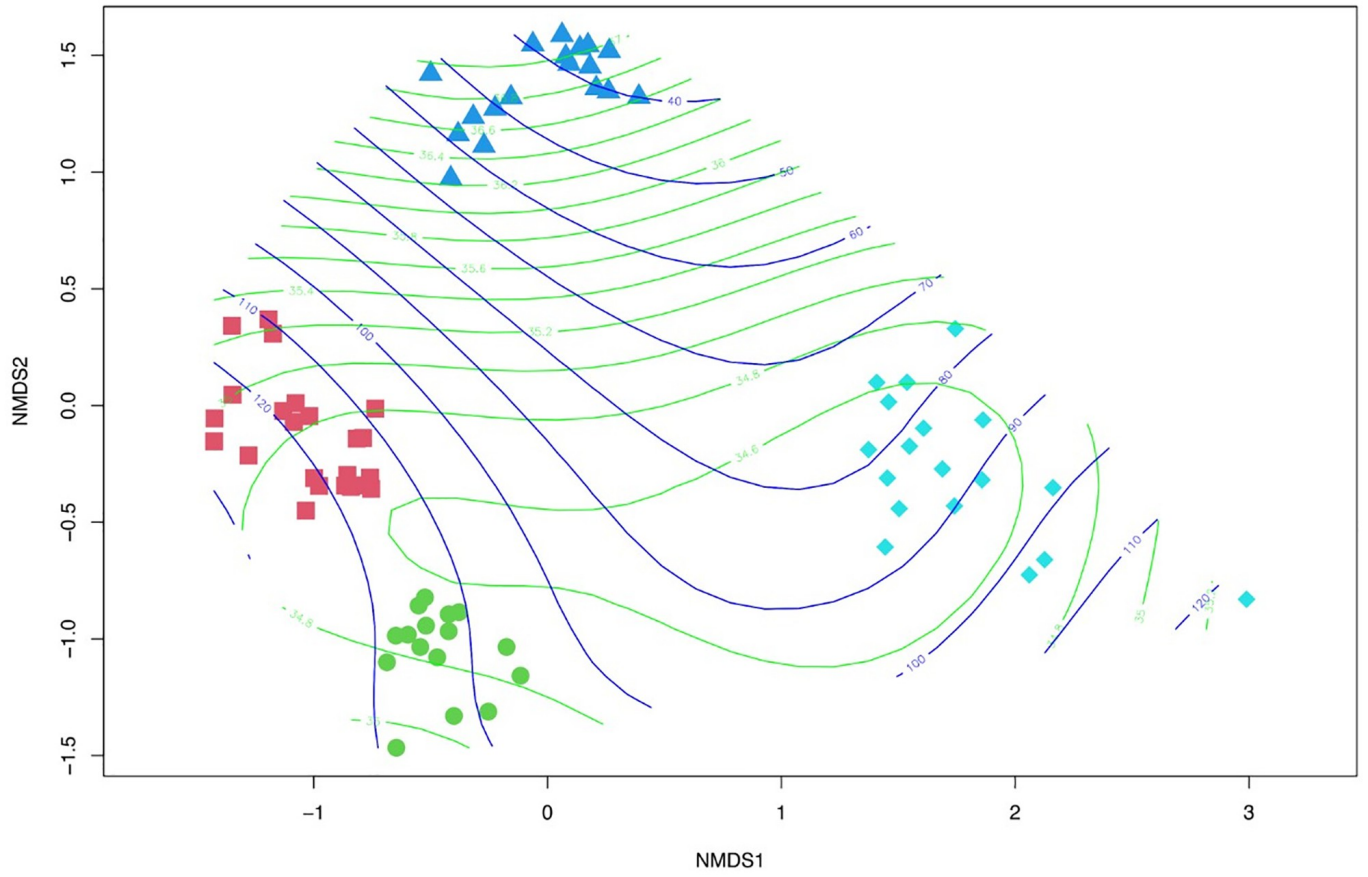
Ordination plot NMDS: Between species comparison. Separation of individuals of each species based on venom profiles (using Bray-Curtis distance). Red squares = *H*. *valida*; green circles = *H*. *infensa*; dark blue triangles = *H*. *cerberea*, and light blue diamonds = *A*. *robustus*. Stress value = 0.148. The lines represent the projection of the significant behavioural (blue: Activity) and morphophysiological (green: Heart rate) variables on the NMSD plot of venom components. Activity decreases as it projects over *H*. *cerberea*. In contrast, heart rate increases as it projects over *H*. *cerberea*, but shows a more homogenous pattern in the other species.

After running the NMDS in the complete venom matrix, we observed that the analyses were not identifying patterns of variation between species due to the higher correlation between venom molecules between species (a high number of components with only 76 individuals). Thus, we used only the reduced venom matrix for these analyses. After performing the Spearman rank correlation, the venom matrix of all species was reduced from 503 to 136 venom components. The NMDS of venom components obtained from all species showed that each species formed a distinct group according to its venom profiles (Adonis, F.model = 21.891, R^2^ = 0.481, P = 0.001, Fig 3). The venom components that might drive the patterns of species distribution in the plot can be observed in the Supplementary material (S4A and S4B Table). When we projected the behavioural and morphophysiological variables into the NMDS plot of venom components, we found that the variation along NMDS1 and NMDS2 was positively correlated with heart rate (NMDS1 = 0.033, NMDS2 = 0.999, r^2^ = 0.125, P = 0.010; Fig 3), and negatively correlated with activity (NMDS1 = -0.210, NMDS2 = -0.977, r^2^ = 0.096, P = 0.023; Fig 3; S4C Table).

## Discussion

The ecological function of venoms in Australian funnel-web spiders is influenced by different behavioural phenotypes that can also be directly or indirectly affected by physiological and morphological traits. This is the first study to assess the synergistic associations between behavioural, physiological and morphological traits in mygalomorphs spiders (funnel-web spiders) using innovative multivariate statistical techniques. We show for the first time how specific venom components are associated with particular behavioural and physiological variables and demonstrate that these relationships are context-dependant.

### Within species

For *H*. *valida*, we found significant associations between venom components, defence behaviour and heart rate. Defensive behaviour was positively associated with heart rate frequency (a proxy of metabolic rate [33]) and also with the expression of specific venom components (i.e., 439, 489 and 4397), which were either positively or negatively associated depending on the ecological context (predation). Although most of the venom molecules in *H*. *valida* have not yet been characterised, two of them have been identified: ω-atracotoxin-Hi1b (4047 Da) and δ-atracotoxin-Hva1b (4702 Da). ω-atracotoxin-Hi1b was negatively correlated with defensive behaviour and is also found in the closely related species *H*. *infensa* [57]. This toxin has insecticidal properties and is highly selective for insect voltage-gated calcium channels [57,58]. Spiders may not produce this toxin during anti-predator defence to reduce energetic costs of costly venom molecule production. δ-atracotoxin-Hva1b was positively associated with heart rate and acts by delaying the inactivation of both vertebrate tetrodotoxin-sensitive voltage-gated sodium (Na_V_) channels and insect para-type sodium channels [57,59]. If this toxin is costly to produce, spiders with a higher metabolic rate may be able to invest more into its production.

Understanding the dynamics of intrinsic and extrinsic factors together with behavioural traits will allow for harvesting/targeting of particular toxin molecules that may lead to novel drug lead discovery. For example, in *Apis mellifera*, the protein profile and weight of bee venom are affected by behavioural responses (degree of aggression) and ecological factors, such as temperature and geographical location, which can affect the venom harvest [60]. However, more work is needed to understand these complex relationships in their entirety.

The association between defence behaviour and heart rate has also been reported in the web-building spider, *Larinioides cornutus*, where bolder and more aggressive spiders had higher heart frequencies than shy spiders [34]. These types of correlations are maintained in animals when abundant resources are available and aggressive behaviours are advantageous for securing resources against conspecifics [34]. The relationships between aggressive behaviours, expression of particular venom components, and heart rates in spiders might depend on the individual’s body condition, mass, age, reproductive state, food intake and environmental conditions [32]. In the case of *H*. *valida*, all the individuals were adult females, showed similar body mass, were under the same environmental conditions, and had the same diet, so these factors are unlikely an explanation for the patterns observed. However, we do not know the age of the females (other than they were all adults), their previous experience in the wild or their genetic relationships, all of which could contribute to the particular relationships we observed.

A relationship between aggression and particular venom components could also be explained by the type of stimulus [27,36]. Spiders can modulate their defensive behaviours according to the type of stimulus or threat to which they are exposed [27]. For example, *A*. *robustus* individuals that were exposed to a prod stimulus showed an increase in the number of fang movements over time [36], and this behaviour is considered a defensive behaviour against predators. Additionally, juveniles and adults responded differently depending on the type of stimulus (puff of air compared to a prod stimulus [36]). A direct stimulus, such as prodding or poking, involves physical contact, which can trigger a cascade of multiple behavioural and physiological changes in individuals, such as increasing the heart rate, and/or neurohormonal changes [61], increasing defensiveness [1,27], and expressing or expelling venom on their fangs [27,36,62]. In contrast, an indirect stimulus, such as a puff of air, could elicit a suite of different responses such as huddling to reduce conspicuousness or fleeing to avoid a predator [36,63].

When considering the other three species, it is possible that an association between morphophysiological traits and venom components was masked by differences between adult and juvenile stages in *H*. *cerberea* and *A*. *robustus*. It has been shown that inter- and intra-individual variation in venom composition over time between adults and juveniles differs independently of diet and environmental conditions [41]. In the case of *H*. *infensa*, it is possible that other morphophysiological traits might also be associated with the use of venom components, but this needs to be addressed in future studies. Furthermore, the behaviour and venom composition of these species is known to change over time, so it would be beneficial to explore whether these associations arise at particular time points within ecological contexts.

### Between species

Interestingly when the venom profiles from all four funnel-web species were compared, we found that venom profiles are a reliable chemotaxonomic marker tool for identifying spider species, even when inter- and intra-individual variation plays an important role as a source of variation in venom components, as has been reported in *H*. *valida* [41]. The use of multivariate statistical approaches thus appears to provide a useful tool for assessing differences between species. However, as other factors, such as genetic structure, ecology, behaviours, geographical variation and environmental conditions can affect the variability of venom components [25], these should all be considered when determining the identification of venomous species.

Surprisingly, despite close taxonomic relationships between *Hadronyche* species, and considerable variation in habits between *H*. *cerberea* and other species (tree- vs. ground-dwellers), we could not clearly differentiate species based on behaviour and morphophysiological traits (or their relationships) in a similar manner to venom composition. Although we did not identify specific relationships between venom components with behavioural and morphophysiological variables in some species, we did observe a significant effect between heart rate and activity with venom profiles from all species when morphophysiological variables were projected on the ordination plot of venoms (NMDS). The correlation between activity and heart rate has also been associated with aggression [32,34], where animals with high metabolic rates are expected to be aggressive, active and bolder to obtain resources to maintain their metabolism [34]. In spiders, venom plays an important role in predation and predatory deterrence, and the behaviours associated with these functions will determine the success or death of the individual [1]. However, the use of venom and the display of aggressive behaviours can lead to metabolic costs [1,13,32]. As a result, spiders might use different behavioural strategies [2] to compensate for these costs. In our results, activity was negatively correlated with venom compounds and positively correlated with heart rate. This suggests that spiders might increase their metabolic rate when they use venoms, and reduce their movement when facing a threat. Other strategies to reduce costs associated with the use of venom include adjusting the number of bites [27], modulating venom deployment and quantity [1], displaying aggressive behaviours without expelling venom [27], and potentially using particular venom molecules when they are exposed to different types of threats, such as predators [1] and conspecifics [36]. For example, spiders can estimate the quantity of venom available in their venom glands, which might help them to select prey [64], and they can also modulate venom expenditure under different threat levels [27]. The relationships between morphophysiological and behavioural traits might have an impact on life history, provoking a change in how animals adapt to changing conditions [32]. The ability of individuals to cope with stressful conditions will determine their ability to survive in different environments [65], such as in places where habitat fragmentation and urbanisation are more evident [66].

Our holistic approach, where multiple traits were included, allowed us to determine the association between particular venom components with behaviours and morphophysiological measurements over different ecological contexts. These results might be considered in antivenom production [67] and the study of bioactive components found in funnel-webs [68], which have a potential for drug discovery [69–72]. Behavioural factors (degree of defence), together with environmental variables, are essential keys to understanding the function and evolution of venoms, as well as their potential as evolutionary models and molecular toolkits in venomous animals.

## Supporting information

S1 Fig Correlation circle plots. a) Correlation circle plots showing the relationship between venom components (complete venom matrix) with behavioural and morphophysiological variables. The variables and venom components outside of the circle show a strong correlation; the variables inside the circle show a weak correlation. b) correlation plot reduced venom matrix Vs venom components. cutoff 0.40.
(TIF)

S2 Fig Network plot reduced venom matrix of a regularised canonical correlation analysis (rCCA). The network plot shows the structure of the association between venom components (reduced venom component matrix) and morphophysiological variables. The correlation cutoff showed is at 0.40. The colour of each line (red: Positive, green: Negative association) indicates the nature of the correlation.
(TIF)

S3 Fig Canonical variates corresponding to the relationship between venom components (complete venom matrix) with behavioural and morphophysiological variables. Individuals are projected into the space spanned by the averaged canonical variates and coloured according to the behavioural and morphophysiological information. a) Defensiveness, complete venom matrix; b) Defence, reduced venom matrix; c) Heart rate, complete venom matrix; d) Heart rate, reduced venom matrix.
(DOCX)

S4 Fig Cluster Image Map from regularised canonical correlation analysis (rCCA) showing the correlation structure of venom components. Complete matrix (a) and reduced matrix (b) with behavioural and morphophysiological variables. The first two dimensions from the CCA are display in the cluster.
(TIF)

S1 Table Output of rank-based non-parametric analyses for longitudinal data models of *H*. *cerberea* and *A*. *robustus*. Test to assess differences in behavioural traits, heart rate and body condition, and the effects of repetitions and life stage. The * refers to results that are significant at the α = 0.05 level.
(DOCX)

S2 Table CCA complete matrix. **(a)** Output axes canonical correspondence analysis (CCA) of complete venom matrix using Chi-square distance Vs behaviour and morphophysiological variables. **(b)** Output axes canonical correspondence analysis (CCA) of complete venom matrix using Bray-Curtis distance Vs behaviour and morphophysiological variables. **(c)** Canonical eigenvalues results are the same for the model with both distances. **(d)** Coefficients CCA using Chi-square distance. **(e)** Coefficients CCA using Bray-Curtis.
(DOCX)

S3 Table CCA reduced matrix. **(a)** Output axes canonical correspondence analysis (CCA) of reduced venom matrix using Chi-square distance Vs morphophysiological variables. **(b)** Output axes canonical correspondence analysis (CCA) of reduced venom matrix using bray-curtis distance Vs morphophysiological variables. **(c)** Coefficients CCA using Chi-square distance. **(d)** Coefficients CCA using bray-curtis.
(DOCX)

S4 Table **(a)** Envfit analysis showing the venom components driving the species distribution pattern. The * refers to results that are significant at the α = 0.05 level. **(b)** Output Anova permutation test showing different variances between species. The * refers to results that are significant at the α = 0.05 level. **(c)** Envfit analysis showing the correlation between behavioural and morphophysiological variables with venom components. The * refers to results that are significant at the α = 0.05 level.
(DOCX)

S1 Appendix Heart rate monitor- description. (DOCX)

S1 File (ZIP)
