# Supplementary figures and images for: Interactions between physiology and behaviour provide insights into the ecological role of venom in Australian funnel-web spiders: Interspecies comparison
Source: PLoS One. 2023 May 22;18(5):e0285866. doi: 10.1371/journal.pone.0285866 (PMC10202279; doi:10.1371/journal.pone.0285866)

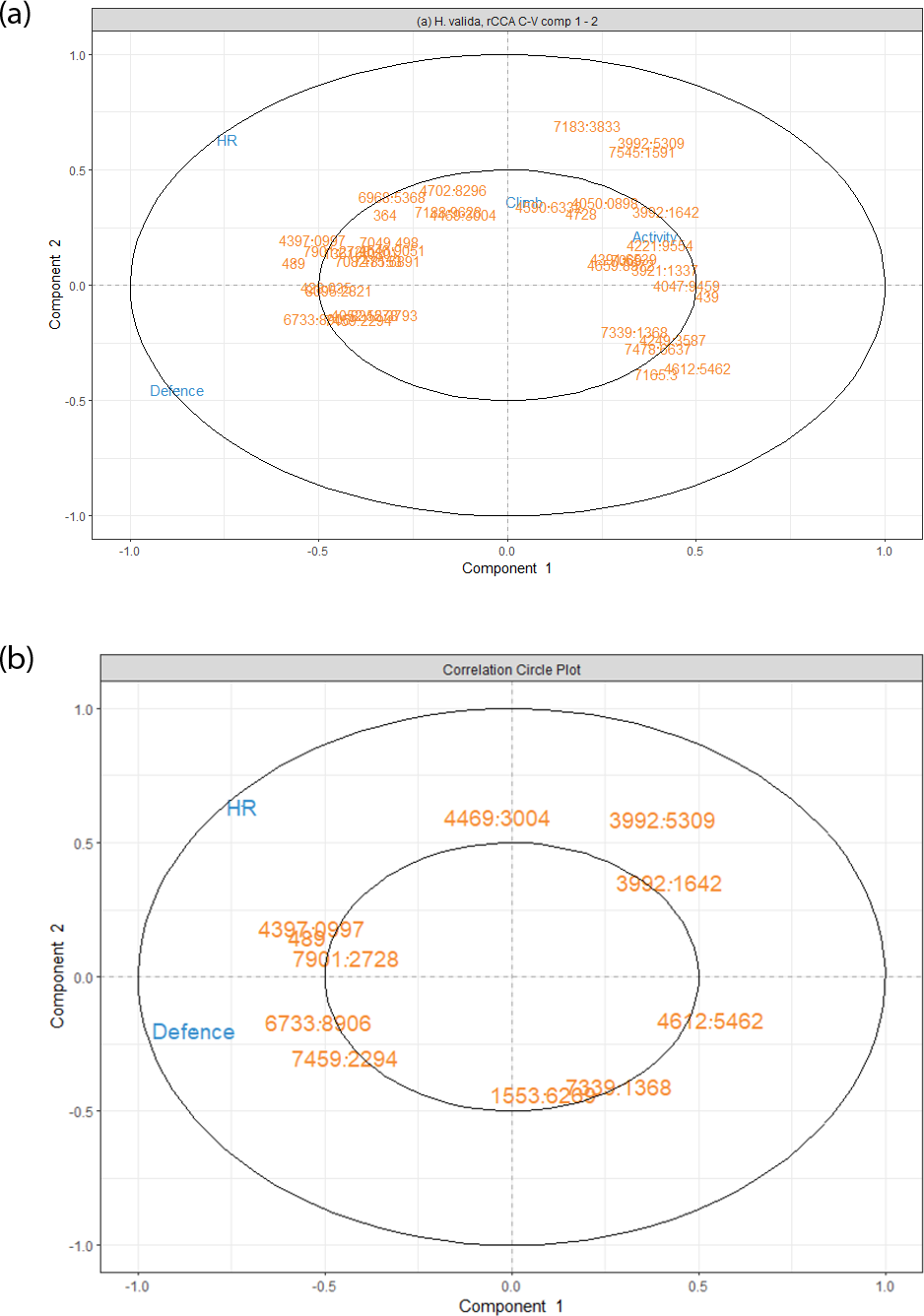

Supplement: S1 Fig — a) Correlation circle plots showing the relationship between venom components (complete venom matrix) with behavioural and morphophysiological variables. The variables and venom components outside of the circle show a strong correlation; the variables inside the circle show a weak correlation. b) correlation plot reduced venom matrix Vs venom components. cutoff 0.40. (TIF) [file pone.0285866.s001.tif]

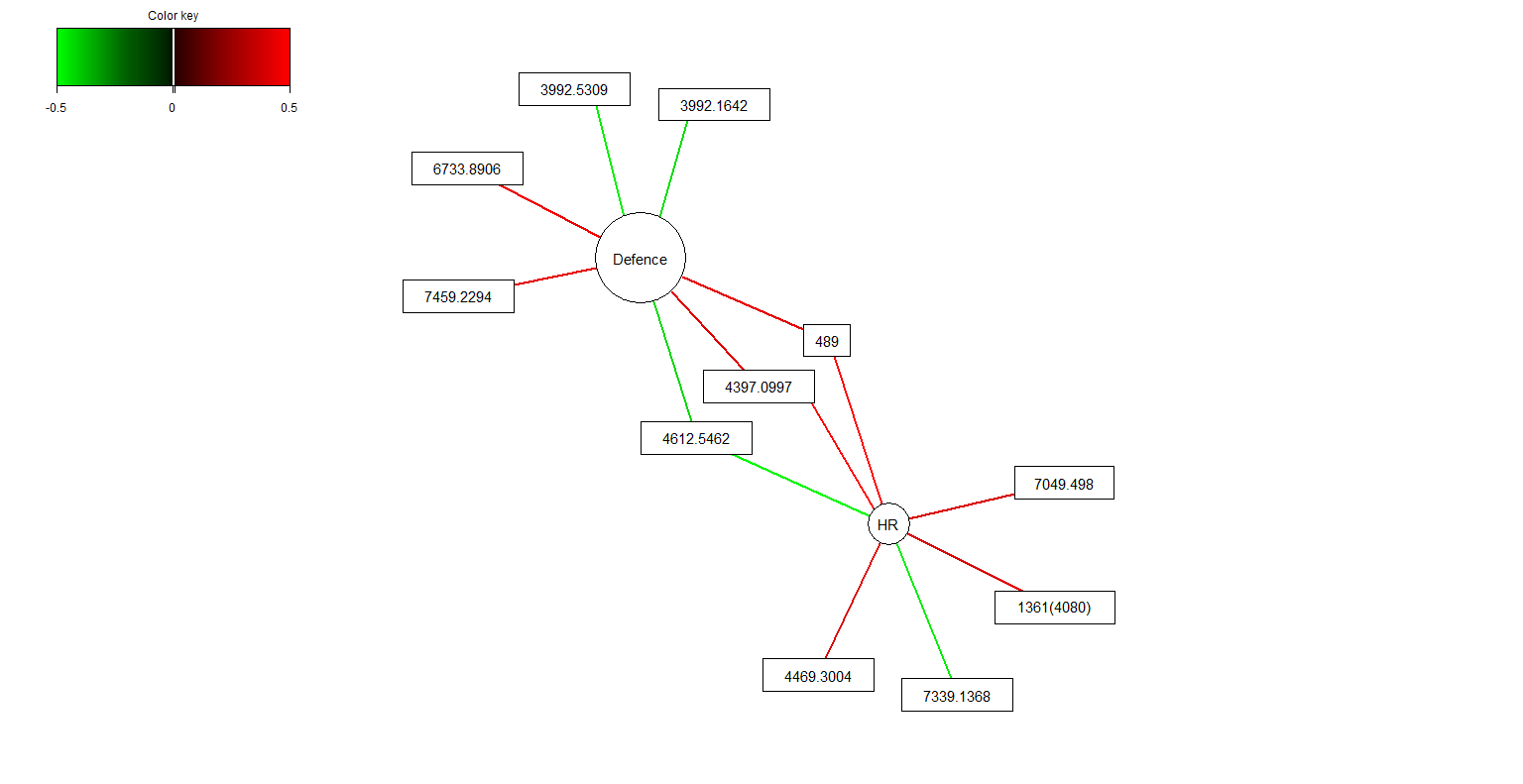

Supplement: S2 Fig — The network plot shows the structure of the association between venom components (reduced venom component matrix) and morphophysiological variables. The correlation cutoff showed is at 0.40. The colour of each line (red: Positive, green: Negative association) indicates the nature of the correlation. (TIF) [file pone.0285866.s002.tif]

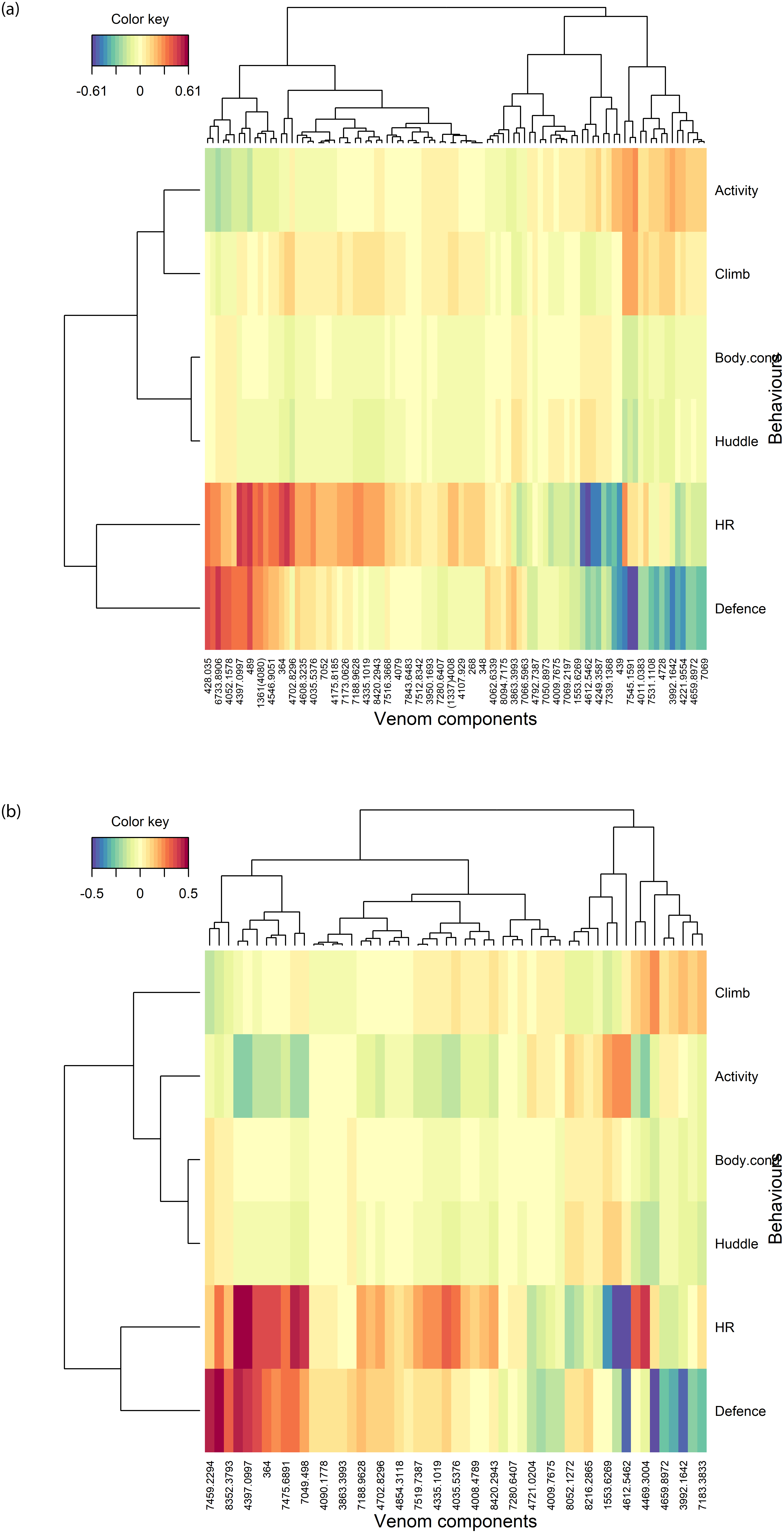

Supplement: S4 Fig — Complete matrix (a) and reduced matrix (b) with behavioural and morphophysiological variables. The first two dimensions from the CCA are display in the cluster. (TIF) [file pone.0285866.s004.tif]
