# Supplementary material for: Interactions between physiology and behaviour provide insights into the ecological role of venom in Australian funnel-web spiders: Interspecies comparison
Source: PLoS One. 2023 May 22;18(5):e0285866. doi: 10.1371/journal.pone.0285866 (PMC10202279; doi:10.1371/journal.pone.0285866)
Supplement: S3 Fig — Individuals are projected into the space spanned by the averaged canonical variates and coloured according to the behavioural and morphophysiological information. a) Defensiveness, complete venom matrix; b) Defence, reduced venom matrix; c) Heart rate, complete venom matrix; d) Heart rate, reduced venom matrix. (DOCX) [file pone.0285866.s003.docx]

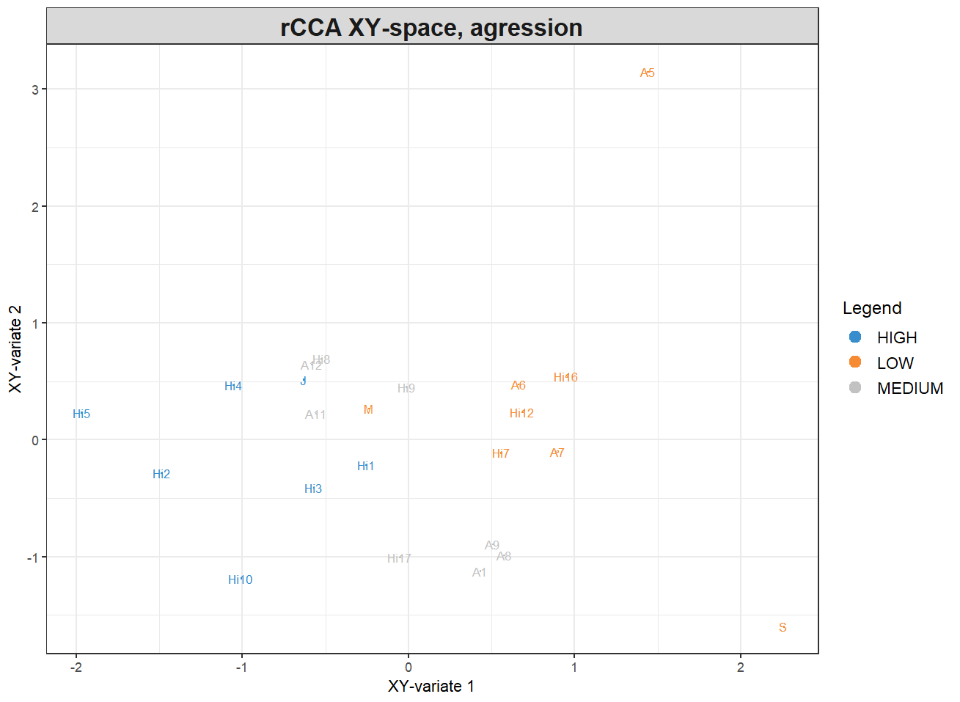

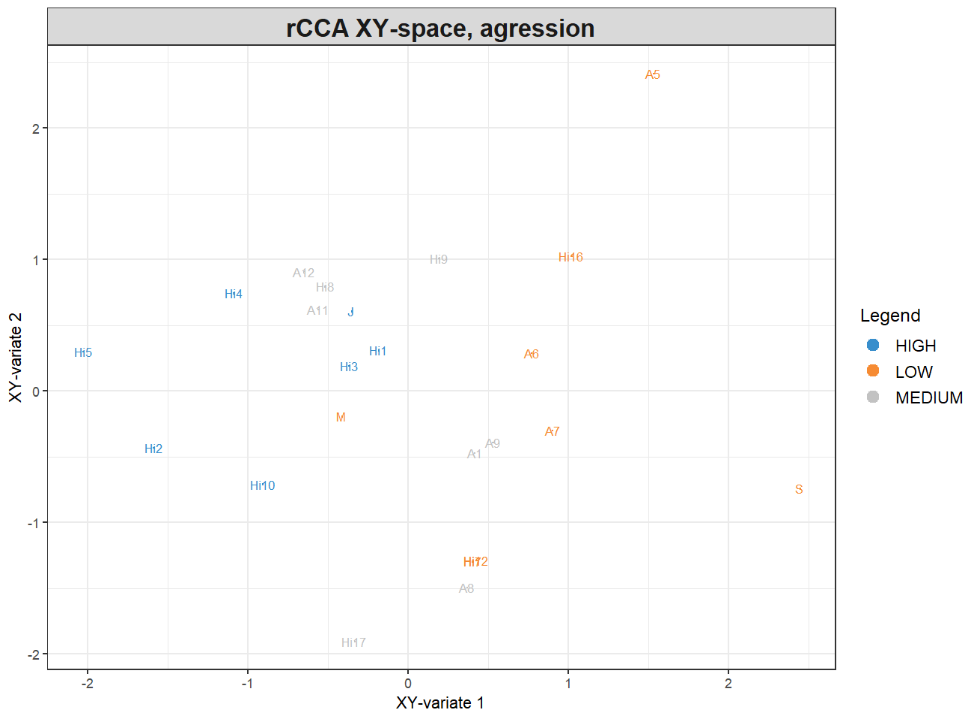
**a. b.**


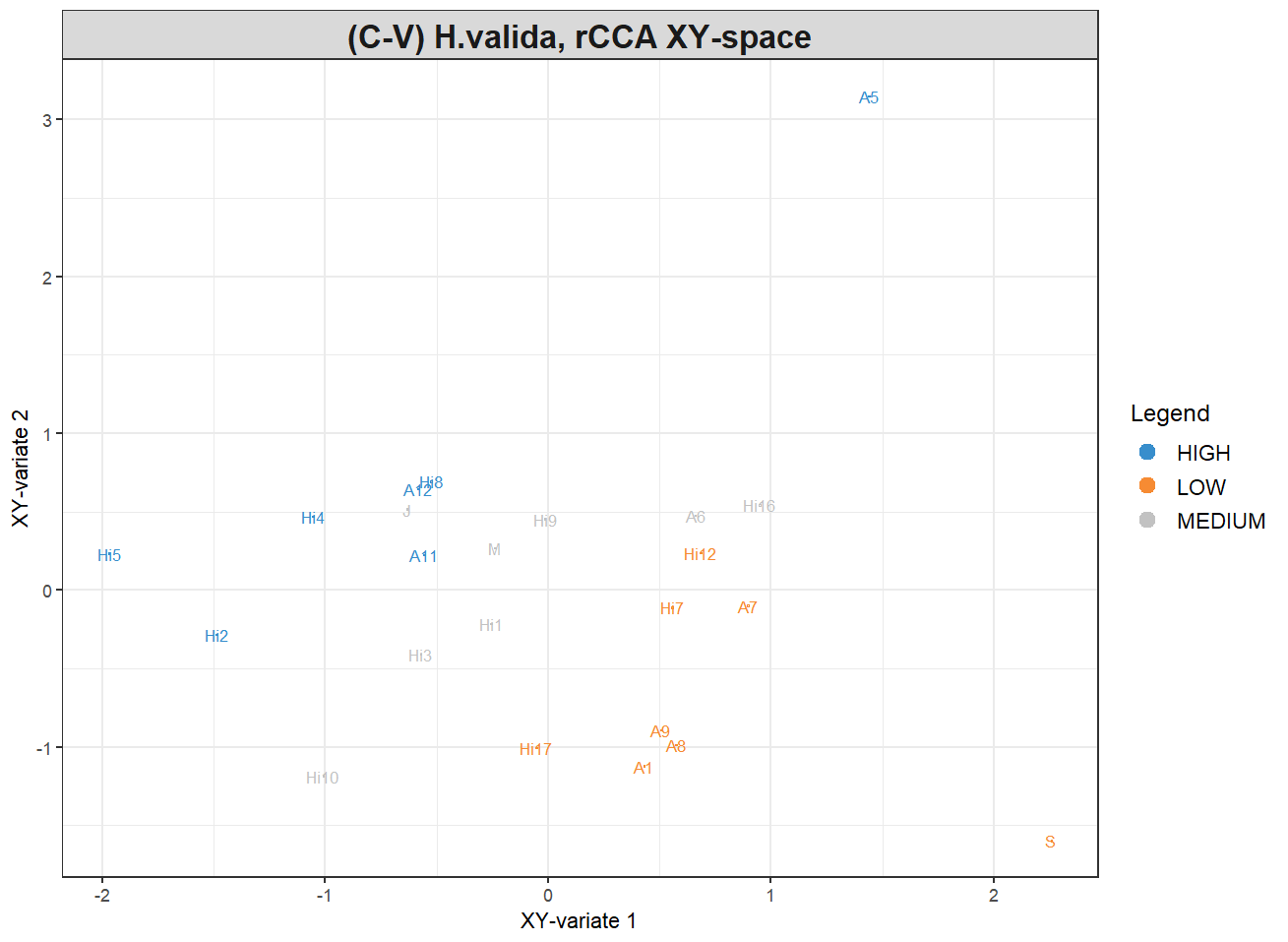

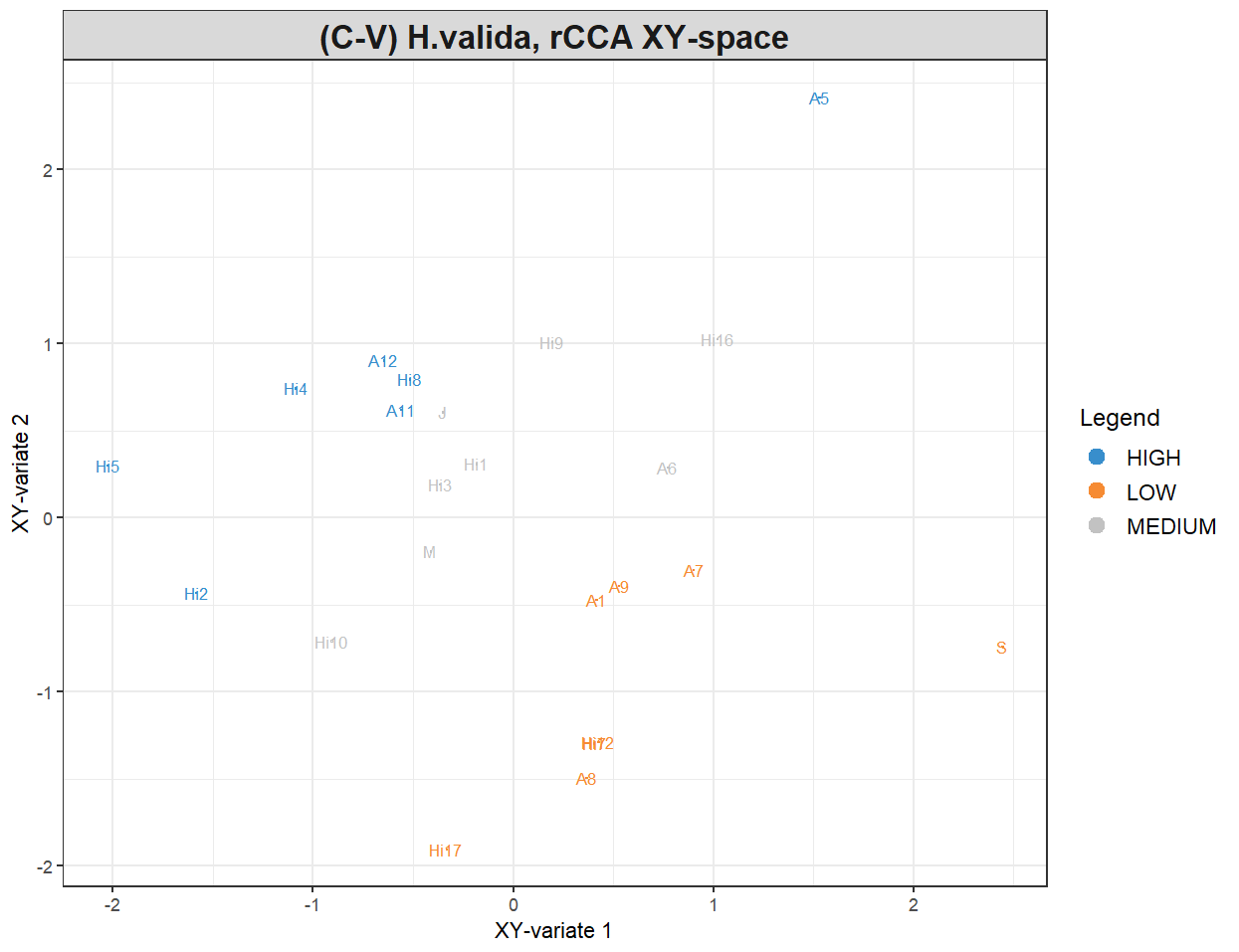
**c. d.**

**S3 Fig.** **Canonical variates corresponding to the relationship between venom components (complete venom matrix) with behavioural and morphophysiological variables**. Individuals are projected into the space spanned by the averaged canonical variates and coloured according to the behavioural and morphophysiological information. a) Defensiveness, complete venom matrix; b) Defence, reduced venom matrix; c) Heart rate, complete venom matrix; d) Heart rate, reduced venom matrix.
