# Supplementary material for: Interactions between physiology and behaviour provide insights into the ecological role of venom in Australian funnel-web spiders: Interspecies comparison
Source: PLoS One. 2023 May 22;18(5):e0285866. doi: 10.1371/journal.pone.0285866 (PMC10202279; doi:10.1371/journal.pone.0285866)
Supplement: S1 Table — Test to assess differences in behavioural traits, heart rate and body condition, and the effects of repetitions and life stage. The * refers to results that are significant at the α = 0.05 level. (DOCX) [file pone.0285866.s005.docx]

**S1** **Table.** **Output of rank-based non-parametric analyses** **for longitudinal data models of *H. cerberea* and *A. robustus*.** Test to assess differences in behavioural traits, heart rate and body condition, and the effects of repetitions and life stage. The * refers to results that are significant at the α = 0.05 level.

| **ANOVA-Type Statistic (ATS)** | | | |  |
| --- | --- | --- | --- | --- |
| **Model** | Huddling ~ Repetition + Stage, subject= factor (ID) | | | |
| *H. cerberea* | | | | |
|  | **Statistic** | **df** | **p-value** |  |
| **Stage** | 0.680 | 1 | 0.421 |  |
| **Repetition** | 0.112 | 1.961 | 0.889 |  |
| *A. robustus* | | | | |
| **Stage** | 2.093 | 1 | 0.147 |  |
| **Repetition** | 3.518 | 1.884 | 0.032 * |  |
| **Model** | Defence (fang frequency ) ~ Repetition + Stage, subject= factor (ID) | | | |
| *H. cerberea* | | | | |
|  | **Statistic** | **df** | **p-value** |  |
| **Stage** | 3.294 | 1 | 0.09 |  |
| **Repetition** | 0.596 | 1.877 | 0.54 |  |
| *A. robustus* | | | | |
| **Stage** | 2.247 | 1 | 0.195 |  |
| **Repetition** | 26.308 | 1.989 | <0.001 * |  |
| **Model** | Defence (frequency of climbs) ~ Repetition + Stage, subject= factor (ID) | | | |
| *H. cerberea* | | | | |
|  | **Statistic** | **df** | **p-value** |  |
| **Stage** | 0.184 | 1 | 0.673 |  |
| **Repetition** | 1.029 | 1.893 | 0.354 |  |
| *A. robustus* | | | | |
| **Stage** | 0.516 | 1 | 0.472 |  |
| **Repetition** | 0.468 | 1.828 | 0.607 |  |
| **Model** | Activity ~ Repetition + Stage, subject= factor (ID) | | | |
| *A. robustus* | | | | |
|  | **Statistic** | **df** | **p-value** |  |
| **Stage** | 0.145 | 1 | 0.703 |  |
| **Repetition** | 1.59 | 1.723 | 0.207 |  |
| **Model** | Heart rate ~ Repetition + Stage, subject= factor (ID) | | | |
| *H. cerberea* | | | | |
|  | **Statistic** | **df** | **p-value** |  |
| **Stage** | 0.006 | 1 | 0.940 |  |
| **Repetition** | 1.094 | 1.9 | 0.332 |  |
| *A. robustus* | | | | |
| **Stage** | 0.042 | 1 | 0.839 |  |
| **Repetition** | 0.300 | 1.564 | 0.685 |  |
| **Model** | Body condition ~ Repetition + Stage, subject= factor (ID) | | | |
| *H. cerberea* | | | | |
|  | **Statistic** | **df** | **p-value** |  |
| **Stage** | 2.131 | 1 | 0.1636 |  |
| **Repetition** | 16.456 | 1.827 | <0.001 * |  |
| *A. robustus* | | | | |
| **Stage** | 0.764 | 1 | 0.410 |  |
| **Repetition** | 2.425 | 1.252 | 0.111 |  |
