# Supplementary material for: Interactions between physiology and behaviour provide insights into the ecological role of venom in Australian funnel-web spiders: Interspecies comparison
Source: PLoS One. 2023 May 22;18(5):e0285866. doi: 10.1371/journal.pone.0285866 (PMC10202279; doi:10.1371/journal.pone.0285866)
Supplement: S2 Table — (a) Output axes canonical correspondence analysis (CCA) of complete venom matrix using Chi-square distance Vs behaviour and morphophysiological variables. (b) Output axes canonical correspondence analysis (CCA) of complete venom matrix using Bray-Curtis distance Vs behaviour and morphophysiological variables. (c) Canonical eigenvalues results are the same for the model with both distances. (d) Coefficients CCA using Chi-square distance. (e) Coefficients CCA using Bray-Curtis. (DOCX) [file pone.0285866.s006.docx]

**S2** **Table.** **CCA complete matrix. (a)** Output axes canonical correspondence analysis (CCA) of complete venom matrix using Chi-square distance Vs behaviour and morphophysiological variables. **(b)** Output axes canonical correspondence analysis (CCA) of complete venom matrix using Bray-Curtis distance Vs behaviour and morphophysiological variables. **(c)** Canonical eigenvalues results are the same for the model with both distances. **(d)** Coefficients CCA using Chi-square distance. **(e)** Coefficients CCA using Bray-Curtis.

**a.**

| **Model** | **Venom matrix ~ Heart rate + body condition + defence + climb + activity + huddle, method = Chi-square** | | | |
| --- | --- | --- | --- | --- |
|  | Df | ChiSquare | F | Pr(>F) |
| CCA1 | 1 | 0.0863 | 2.7375 | 0.408 |
| CCA2 | 1 | 0.06361 | 2.0177 | 0.530 |
| CCA3 | 1 | 0.04743 | 1.5045 | 0.697 |
| CCA4 | 1 | 0.034 | 1.0785 | 0.856 |
| CCA5 | 1 | 0.02794 | 0.8862 | 0.817 |
| CCA6 | 1 | 0.01608 | 0.51 | 0.903 |
| Residual | 16 | 0.5044 |  |  |

**b.**

| **Model:** | **Venom matrix ~ heart rate + body condition + defence + climb + activity + huddle, method = Bray-Curtis** | | | |
| --- | --- | --- | --- | --- |
|  | Df | Bray-curtis | F | Pr(>F) |
| CCA1 | 1 | 0.0863 | 2.7375 | 0.365 |
| CCA2 | 1 | 0.06361 | 2.0177 | 0.464 |
| CCA3 | 1 | 0.04743 | 1.5045 | 0.642 |
| CCA4 | 1 | 0.034 | 1.0785 | 0.83 |
| CCA5 | 1 | 0.02794 | 0.8862 | 0.8 |
| CCA6 | 1 | 0.01608 | 0.51 | 0.903 |
| Residual | 16 | 0.5044 |  |  |

**c.**

|  |  |  |  |  |  |  |  |  |  |
| --- | --- | --- | --- | --- | --- | --- | --- | --- | --- |
|  | **CCA1** | **CCA2** | **CCA3** | **CCA4** | **CCA5** | **CCA6** | **CA1** | **CA2** | **CA3** |
| Eigenvalue | 0.086 | 0.064 | 0.047 | 0.034 | 0.028 | 0.016 | 0.181 | 0.079 | 0.044 |
| Proportion explained | 0.111 | 0.082 | 0.061 | 0.044 | 0.036 | 0.021 | 0.232 | 0.101 | 0.056 |
| Cumulative proportion | 0.111 | 0.192 | 0.253 | 0.297 | 0.333 | 0.353 | 0.585 | 0.686 | 0.743 |
|  | **CA4** | **CA5** | **CA6** | **CA7** | **CA8** | **CA9** | **CA10** | **CA11** | **CA12** |
| Eigenvalue | 0.039 | 0.030 | 0.021 | 0.019 | 0.017 | 0.015 | 0.013 | 0.012 | 0.011 |
| Proportion explained | 0.050 | 0.038 | 0.028 | 0.024 | 0.022 | 0.019 | 0.017 | 0.016 | 0.014 |
| Cumulative proportion | 0.793 | 0.831 | 0.859 | 0.883 | 0.904 | 0.923 | 0.940 | 0.956 | 0.970 |
|  | **CA13** | **CA14** | **CA15** | **CA16** |  |  |  |  |  |
| Eigenvalue | 0.008 | 0.007 | 0.004 | 0.003 |  |  |  |  |  |
| Proportion explained | 0.011 | 0.009 | 0.006 | 0.004 |  |  |  |  |  |
| Cumulative proportion | 0.981 | 0.990 | 0.996 | 1 |  |  |  |  |  |

**d.**

|  | **CCA1** | **CCA2** | **CCA3** | **CCA4** | **CCA5** | **CCA6** |
| --- | --- | --- | --- | --- | --- | --- |
| Heart rate | -0.437 | -0.022 | 0.311 | 0.008 | 0.120 | 0.134 |
| Body condition | -0.102 | -2.584 | 0.434 | -3.665 | 1.402 | 0.042 |
| Defence | 0.003 | 0.077 | -0.032 | -0.048 | -0.024 | -0.020 |
| Climb | 0.445 | 0.236 | 0.300 | -0.004 | 0.312 | 0.009 |
| Activity | -0.002 | 0.002 | 0.003 | -0.004 | -0.007 | 0.000 |
| Huddle | -1.527 | -0.564 | 1.158 | 1.304 | 0.152 | -2.509 |

**e.**

|  | **CCA1** | **CCA2** | **CCA3** | **CCA4** | **CCA5** | **CCA6** |
| --- | --- | --- | --- | --- | --- | --- |
| Body condition | -0.102 | -2.584 | 0.434 | -3.665 | 1.402 | 0.042 |
| Heart rate | -0.437 | -0.022 | 0.311 | 0.008 | 0.120 | 0.134 |
| Defence | 0.003 | 0.077 | -0.032 | -0.048 | -0.024 | -0.020 |
| Climb | 0.445 | 0.236 | 0.300 | -0.004 | 0.312 | 0.009 |
| Activity | -0.002 | 0.002 | 0.003 | -0.004 | -0.007 | 0.000 |
| Huddle | -1.527 | -0.564 | 1.158 | 1.304 | 0.152 | -2.509 |
