# Supplementary material for: Interactions between physiology and behaviour provide insights into the ecological role of venom in Australian funnel-web spiders: Interspecies comparison
Source: PLoS One. 2023 May 22;18(5):e0285866. doi: 10.1371/journal.pone.0285866 (PMC10202279; doi:10.1371/journal.pone.0285866)
Supplement: S3 Table — (a) Output axes canonical correspondence analysis (CCA) of reduced venom matrix using Chi-square distance Vs morphophysiological variables. (b) Output axes canonical correspondence analysis (CCA) of reduced venom matrix using bray-curtis distance Vs morphophysiological variables. (c) Coefficients CCA using Chi-square distance. (d) Coefficients CCA using bray-curtis. (DOCX) [file pone.0285866.s007.docx]

**S3** **Table. CCA reduced matrix.** **(a)** Output axes canonical correspondence analysis (CCA) of reduced venom matrix using Chi-square distance Vs morphophysiological variables. **(b)** Output axes canonical correspondence analysis (CCA) of reduced venom matrix using bray-curtis distance Vs morphophysiological variables. **(c)** Coefficients CCA using Chi-square distance. **(d)** Coefficients CCA using bray-curtis.

**a.**

| **Model** | **Reduced venom matrix ~ heart rate + body condition + Defence + climb + activity + huddle** | | | |
| --- | --- | --- | --- | --- |
|  | Df | ChiSquare | F | Pr(>F) |
| CCA1 | 1 | 0.075 | 2.091 | 0.418 |
| CCA2 | 1 | 0.070 | 1.972 | 0.374 |
| CCA3 | 1 | 0.061 | 1.710 | 0.433 |
| CCA4 | 1 | 0.048 | 1.340 | 0.616 |
| CCA5 | 1 | 0.041 | 1.161 | 0.572 |
| CCA6 | 1 | 0.016 | 0.456 | 0.977 |
| Residual | 16 | 0.570 |  |  |

**b.**

| **model:** | **Reduced venom matrix ~ heart rate + body condition + defence + climb + activity + huddle, model = bray** | | | |
| --- | --- | --- | --- | --- |
|  | Df | ChiSquare | F | Pr(>F) |
| CCA1 | 1 | 0.075 | 2.091 | 0.412 |
| CCA2 | 1 | 0.070 | 1.972 | 0.371 |
| CCA3 | 1 | 0.061 | 1.710 | 0.400 |
| CCA4 | 1 | 0.048 | 1.340 | 0.596 |
| CCA5 | 1 | 0.041 | 1.161 | 0.578 |
| CCA6 | 1 | 0.016 | 0.456 | 0.977 |
| Residual | 16 | 0.570 |  |  |

**c.**

|  | **CCA1** | **CCA2** | **CCA3** | **CCA4** | **CCA5** | **CCA6** |
| --- | --- | --- | --- | --- | --- | --- |
| Body condition | -1.639 | -0.245 | 2.167 | 2.865 | 2.633 | 0.505 |
| Heart rate | -0.159 | -0.543 | -0.084 | -0.011 | 0.127 | -0.102 |
| Defence | 0.088 | -0.007 | 0.038 | 0.010 | -0.017 | 0.019 |
| Climb | 0.290 | 0.178 | -0.420 | 0.094 | 0.416 | 0.093 |
| Huddle | -1.369 | -1.634 | -1.166 | -0.219 | -1.014 | 2.262 |
| Activity | 0.003 | -0.004 | 0.001 | 0.005 | -0.004 | -0.002 |

**d.**

|  | **CCA1** | **CCA2** | **CCA3** | **CCA4** | **CCA5** | **CCA6** |
| --- | --- | --- | --- | --- | --- | --- |
| Body condition | -1.639 | -0.245 | 2.167 | 2.865 | 2.633 | 0.505 |
| Heart rate | -0.159 | -0.543 | -0.084 | -0.011 | 0.127 | -0.102 |
| Defence | 0.088 | -0.007 | 0.038 | 0.010 | -0.017 | 0.019 |
| Climb | 0.290 | 0.178 | -0.420 | 0.094 | 0.416 | 0.093 |
| Huddle | -1.369 | -1.634 | -1.166 | -0.219 | -1.014 | 2.262 |
| Activity | 0.003 | -0.004 | 0.001 | 0.005 | -0.004 | -0.002 |
