# Supplementary material for: Interactions between physiology and behaviour provide insights into the ecological role of venom in Australian funnel-web spiders: Interspecies comparison
Source: PLoS One. 2023 May 22;18(5):e0285866. doi: 10.1371/journal.pone.0285866 (PMC10202279; doi:10.1371/journal.pone.0285866)
Supplement: S4 Table — (a) Envfit analysis showing the venom components driving the species distribution pattern. The * refers to results that are significant at the α = 0.05 level. (b) Output Anova permutation test showing different variances between species. The * refers to results that are significant at the α = 0.05 level. (c) Envfit analysis showing the correlation between behavioural and morphophysiological variables with venom components. The * refers to results that are significant at the α = 0.05 level. (DOCX) [file pone.0285866.s008.docx]

**S4** **Table.** **(a)** Envfit analysis showing the venom components driving the species distribution pattern. The * refers to results that are significant at the α = 0.05 level. **(b)** Output Anova permutation test showing different variances between species. The * refers to results that are significant at the α = 0.05 level. **(c)** Envfit analysis showing the correlation between behavioural and morphophysiological variables with venom components. The * refers to results that are significant at the α = 0.05 level.

**a.**

| **Venom components** | **NMDS1** | **NMDS2** | **r2** | **P. value** | |
| --- | --- | --- | --- | --- | --- |
| 489 | -0.99309 | -0.11733 | 0.131 | 0.01 | ** |
| 268 | -0.98441 | 0.17588 | 0.4234 | 0.001 | *** |
| 279 | -0.99462 | 0.1036 | 0.3611 | 0.001 | *** |
| 3863.3993 | -0.96671 | -0.25587 | 0.1833 | 0.004 | ** |
| 7843.6483 | -0.99152 | -0.12999 | 0.0578 | 0.098 | . |
| 3992.5309 | -0.70753 | -0.70669 | 0.0629 | 0.086 | . |
| 3921.1337 | -0.49235 | -0.8704 | 0.448 | 0.001 | *** |
| 4035.5376 | -0.95872 | -0.28435 | 0.05 | 0.153 |  |
| 3950.1693 | -0.99695 | -0.07801 | 0.1155 | 0.01 | ** |
| 4056 | -0.97706 | -0.21297 | 0.0381 | 0.22 |  |
| 7082.8153 | -0.99834 | 0.05766 | 0.1257 | 0.005 | ** |
| 7066.5963 | -0.98337 | -0.18163 | 0.2302 | 0.001 | *** |
| 7049.498 | -0.96049 | -0.27831 | 0.2394 | 0.002 | ** |
| 4854.3118 | -0.99075 | -0.13567 | 0.0189 | 0.574 |  |
| 4608.3235 | -0.99184 | -0.12749 | 0.1648 | 0.004 | ** |
| 7545.1591 | -0.90463 | -0.4262 | 0.0443 | 0.171 |  |
| 4840.0408 | -0.97042 | -0.24141 | 0.0136 | 0.719 |  |
| 1553.6269 | -0.99501 | -0.09974 | 0.0436 | 0.194 |  |
| 4011.0383 | -0.89166 | -0.4527 | 0.0728 | 0.061 | . |
| 4612.5462 | -0.8727 | -0.48825 | 0.0084 | 0.925 |  |
| 8216.2865 | -0.99622 | 0.08681 | 0.1404 | 0.007 | ** |
| 4792.7387 | -0.94 | -0.34117 | 0.0833 | 0.037 | * |
| 7188.9628 | -0.98017 | -0.19815 | 0.1603 | 0.001 | *** |
| 7516.3668 | -0.99518 | -0.09811 | 0.2145 | 0.002 | ** |
| 4163.9178 | -0.99436 | -0.10608 | 0.1242 | 0.008 | ** |
| 7459.2294 | -0.9835 | -0.18091 | 0.1042 | 0.01 | ** |
| 7512.8342 | -0.96413 | -0.26544 | 0.0066 | 0.983 |  |
| 7475.6891 | -0.99749 | -0.07083 | 0.0483 | 0.134 |  |
| 8352.3793 | -0.99567 | 0.09299 | 0.0915 | 0.029 | * |
| 7339.1368 | -0.94489 | -0.3274 | 0.0617 | 0.084 | . |
| 8420.2943 | -0.61494 | -0.78857 | 0.598 | 0.001 | *** |
| 5216.0996 | -0.28488 | -0.95856 | 0.1159 | 0.01 | ** |
| 500.3815 | -0.21426 | -0.97678 | 0.1732 | 0.002 | ** |
| 4052.4924 | -0.26475 | -0.96432 | 0.0463 | 0.165 |  |
| 7843 | -0.24653 | -0.96914 | 0.1079 | 0.014 | * |
| 7798 | -0.23584 | -0.97179 | 0.0374 | 0.262 |  |
| 3724.2321 | -0.23463 | -0.97208 | 0.0806 | 0.043 | * |
| 8114.0185 | -0.11272 | -0.99363 | 0.0349 | 0.235 |  |
| 414 | -0.28409 | -0.9588 | 0.0876 | 0.032 | * |
| 4217.0134 | -0.19417 | -0.98097 | 0.2277 | 0.002 | ** |
| 7057.6814 | -0.24257 | -0.97013 | 0.0607 | 0.085 | . |
| 7139.2911 | -0.17727 | -0.98416 | 0.1049 | 0.019 | * |
| 7043.5521 | -0.29569 | -0.95529 | 0.089 | 0.027 | * |
| 8036.0348 | -0.29833 | -0.95446 | 0.0403 | 0.18 |  |
| 4824.5909 | -0.21716 | -0.97613 | 0.0845 | 0.036 | * |
| 8160.2386 | -0.29671 | -0.95497 | 0.0186 | 0.654 |  |
| 7564.274 | -0.21957 | -0.9756 | 0.0176 | 0.68 |  |
| 7172.9124 | -0.18175 | -0.98334 | 0.1646 | 0.005 | ** |
| 7429.1389 | -0.12275 | -0.99244 | 0.0802 | 0.038 | * |
| 4202.8985 | -0.2273 | -0.97383 | 0.1948 | 0.001 | *** |
| 4859.7366 | -0.24436 | -0.96969 | 0.1693 | 0.001 | *** |
| 7295.1263 | -0.13951 | -0.99022 | 0.0409 | 0.14 |  |
| 6793.0155 | -0.22258 | -0.97491 | 0.0357 | 0.232 |  |
| 7175.1997 | -0.21919 | -0.97568 | 0.2941 | 0.001 | *** |
| 1336.1044 | -0.15472 | -0.98796 | 0.1818 | 0.001 | *** |
| 298.0852 | -0.05079 | 0.99871 | 0.2959 | 0.001 | *** |
| 261.2227 | -0.05161 | 0.99867 | 0.3121 | 0.001 | *** |
| 373.7885 | -0.03735 | 0.9993 | 0.4129 | 0.001 | *** |
| 330.1327 | -0.06424 | 0.99793 | 0.0411 | 0.174 |  |
| 394.0119 | -0.18397 | 0.98293 | 0.0677 | 0.052 | . |
| 694.2625 | -0.09694 | 0.99529 | 0.1429 | 0.005 | ** |
| 617.4858 | -0.00224 | 1 | 0.0422 | 0.097 | . |
| 2562.3652 | -0.10624 | 0.99434 | 0.2667 | 0.001 | *** |
| 428.1218 | -0.19285 | 0.98123 | 0.0783 | 0.029 | * |
| 1469.4387 | -0.2604 | 0.9655 | 0.0333 | 0.254 |  |
| 292.7969 | -0.24091 | 0.97055 | 0.0414 | 0.157 |  |
| 6670.4303 | 0.10839 | 0.99411 | 0.1267 | 0.006 | ** |
| 3906.4078 | -0.27901 | 0.96029 | 0.0176 | 0.696 |  |
| 3659.8361 | -0.01625 | 0.99987 | 0.0823 | 0.037 | * |
| 4877.4044 | -0.01688 | 0.99986 | 0.2424 | 0.001 | *** |
| 8168.6186 | -0.06341 | 0.99799 | 0.3355 | 0.001 | *** |
| 7827.0282 | -0.10076 | 0.99491 | 0.114 | 0.011 | * |
| 3911.6057 | -0.08416 | 0.99645 | 0.1856 | 0.001 | *** |
| 7827.0419 | 0.18659 | 0.98244 | 0.0394 | 0.152 |  |
| 7514.7821 | -0.05261 | 0.99862 | 0.4009 | 0.001 | *** |
| 4604.1502 | -0.02446 | 0.9997 | 0.1332 | 0.007 | ** |
| 3392.005 | -0.04071 | 0.99917 | 0.2824 | 0.001 | *** |
| 4231.4868 | 0.00143 | 1 | 0.4325 | 0.001 | *** |
| 4135.8916 | -0.07916 | 0.99686 | 0.0908 | 0.025 | * |
| 4174.3106 | -0.1265 | 0.99197 | 0.0201 | 0.612 |  |
| 7085.8057 | 0.05802 | 0.99832 | 0.0764 | 0.035 | * |
| 8261.7955 | -0.05834 | 0.9983 | 0.1006 | 0.016 | * |
| 274 | -0.06974 | 0.99756 | 0.2149 | 0.001 | *** |
| 4166 | -0.05562 | 0.99845 | 0.3199 | 0.001 | *** |
| 337.245 | 0.99465 | -0.10328 | 0.0272 | 0.389 |  |
| 351.2583 | 0.99899 | -0.04496 | 0.2171 | 0.001 | *** |
| 298.711 | 0.99781 | -0.06613 | 0.3489 | 0.001 | *** |
| 4840.1894 | 0.99961 | 0.0278 | 0.0735 | 0.044 | * |
| 348(2083.05) | 0.91057 | -0.41336 | 0.1901 | 0.002 | ** |
| 3503.6014 | 0.97846 | -0.20642 | 0.2002 | 0.001 | *** |
| 474.9455 | 0.98759 | -0.15708 | 0.0699 | 0.055 | . |
| 282 | 0.90137 | -0.43305 | 0.2817 | 0.001 | *** |
| 3881.2675 | 0.99989 | -0.01461 | 0.0289 | 0.355 |  |
| 545.3817 | 0.98372 | -0.1797 | 0.1849 | 0.001 | *** |
| 4506.9104 | 0.95265 | -0.30407 | 0.0965 | 0.015 | * |
| 3795.305 | 0.93645 | -0.3508 | 0.3985 | 0.001 | *** |
| 6166.2588 | 0.93606 | -0.35183 | 0.1624 | 0.004 | ** |
| 4744.134 | 0.97411 | 0.22606 | 0.1217 | 0.005 | ** |
| 1561.0635 | 0.90993 | 0.41477 | 0.07 | 0.043 | * |
| 306 | 0.92302 | -0.38476 | 0.2099 | 0.002 | ** |
| 7154.7684 | 0.98135 | -0.19225 | 0.1865 | 0.002 | ** |
| 1460 | 0.89194 | -0.45215 | 0.1564 | 0.001 | *** |
| 4827.471 | 0.97316 | -0.23014 | 0.0633 | 0.072 | . |
| 4826.3111 | 0.97184 | -0.23564 | 0.1449 | 0.005 | ** |
| 4547.8507 | 0.96315 | 0.26897 | 0.0236 | 0.481 |  |
| 7107.6201 | 0.99994 | 0.01094 | 0.1273 | 0.004 | ** |
| 4588.0007 | 0.9871 | -0.16012 | 0.0847 | 0.033 | * |
| 7979.2666 | 0.95486 | -0.29704 | 0.1896 | 0.001 | *** |
| 7156.1833 | 0.96348 | -0.26778 | 0.0613 | 0.075 | . |
| 4185.3277 | 0.88844 | -0.459 | 0.1339 | 0.004 | ** |
| 8125.9065 | 0.80253 | -0.59661 | 0.0475 | 0.071 | . |
| 7963.7815 | 0.90008 | -0.43572 | 0.2598 | 0.001 | *** |
| 7117.4818 | 0.99967 | -0.02575 | 0.1876 | 0.001 | *** |
| 4214.5417 | 0.85928 | -0.51151 | 0.1188 | 0.009 | ** |
| 4547.2444 | 0.98969 | -0.14319 | 0.2537 | 0.001 | *** |
| 7946.7021 | 0.88164 | -0.47193 | 0.2127 | 0.001 | *** |
| 4103.3168 | 0.89521 | -0.44564 | 0.0298 | 0.336 |  |
| 4528.6558 | 0.95237 | -0.30495 | 0.2689 | 0.001 | *** |
| 7229.2298 | 0.92558 | -0.37855 | 0.2115 | 0.001 | *** |
| 4527.9452 | 0.94447 | -0.3286 | 0.0779 | 0.048 | * |
| 7301.7331 | 0.92725 | -0.37445 | 0.0255 | 0.455 |  |
| 7274.4236 | 0.9737 | 0.22782 | 0.1403 | 0.004 | ** |
| 7330.3427 | 0.98583 | 0.16773 | 0.1743 | 0.001 | *** |
| 8821.6224 | 0.94402 | -0.32988 | 0.165 | 0.004 | ** |
| 7299.3702 | 0.99856 | 0.0537 | 0.0365 | 0.191 |  |
| 7618.7917 | 0.89995 | -0.436 | 0.208 | 0.001 | *** |
| 7235.0821 | 0.89329 | -0.44949 | 0.3762 | 0.001 | *** |
| 4606.597(1448.051) | 0.87257 | -0.4885 | 0.0903 | 0.017 | * |
| 7287.73 | 0.96886 | -0.24763 | 0.027 | 0.411 |  |
| 2961.7804 | 0.87322 | -0.48733 | 0.1227 | 0.014 | * |
| 4092.106(1347.312) | 0.96733 | -0.25352 | 0.1863 | 0.001 | *** |
| 1337.3453 | 0.9459 | -0.32447 | 0.2948 | 0.001 | *** |
| 3992.89(1331.195) | 0.89604 | -0.44398 | 0.366 | 0.001 | *** |
| 1394 | 0.87779 | -0.47905 | 0.0344 | 0.251 |  |
| 4076.17(1341) | 0.98958 | -0.14396 | 0.3191 | 0.001 | *** |
| 1384 | 0.8289 | -0.5594 | 0.0185 | 0.637 |  |

**b.**

| **Analysis of variance** | | | | | |
| --- | --- | --- | --- | --- | --- |
|  | Df | Sum Sq | Mean Sq | F value | P. value |
| Groups | 3 | 0.23529 | 0.07843 | 17.912 | < 0.001 |
| Residuals | 71 | 0.31088 | 0.004379 |  |  |
| **Pairwise comparisons between species** | | | | | |
|  |  | *A. robustus* | *H. cerberea* | *H. infensa* | *H. valida* |
| *A. robustus* | |  | 1.00E-03 | 1.00E-03 | 0.001 |
| *H. cerberea* | | 9.26E-06 |  | 5.32E-01 | 0.038 |
| *H. infensa* | | 7.18E-07 | 5.48E-01 |  | 0.136 |
| *H. valida* |  | 3.10E-08 | 4.14E-02 | 1.33E-01 |  |

**c.**

|  |  |  |  |  |  |
| --- | --- | --- | --- | --- | --- |
| **Behaviour** | **NMDS1** | **NMDS2** | **r2** | **P. value** |  |
| Heart rate | 0.03338 | 0.99944 | 0.1256 | 0.01 | ** |
| Defence | -0.30979 | -0.95081 | 0.0439 | 0.163 |  |
| Climb | -0.82594 | -0.56376 | 0.0251 | 0.434 |  |
| Activity | -0.21072 | -0.97755 | 0.0965 | 0.016 | * |
| Huddling | -0.21746 | 0.97607 | 0.0426 | 0.219 |  |
| Body condition | -0.97363 | -0.22811 | 0.0047 | 0.855 |  |
