# Supplementary material for: Interactions between physiology and behaviour provide insights into the ecological role of venom in Australian funnel-web spiders: Interspecies comparison
Source: PLoS One. 2023 May 22;18(5):e0285866. doi: 10.1371/journal.pone.0285866 (PMC10202279; doi:10.1371/journal.pone.0285866)

**S1 Appendix.** **Heart rate monitor- description.**

The signal of the sensor was digitised to facilitate analysis and graphing using MATLAB (model: R2018a, version: 9.4.0.813654). The step of data acquisition implements FIFO (First in First out), where a rapid read of the data is implemented to avoid losing data. A micro-controller was connected to ensure both, the computer and the sensor, were always connected and synchronised to ensure that a clear track for data were present [45]. The only power for the sensor to switch on is 2-wire, Serial Data (SDA) and Serial Clock (SCL). Serial Data are data read through a serial connection, while the Serial Clock gives each data section on the SDA line a specific timing for it to be transferred. This facilitates no overlapping or loss of data [68]. After acquiring the data and securing its integrity, the data were converted into presentable information associated with each spider.

The scrip used to run registered the heart rate monitor using MATAB is described below:

Open and run


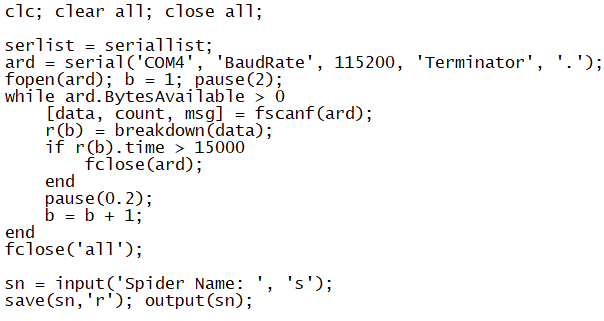


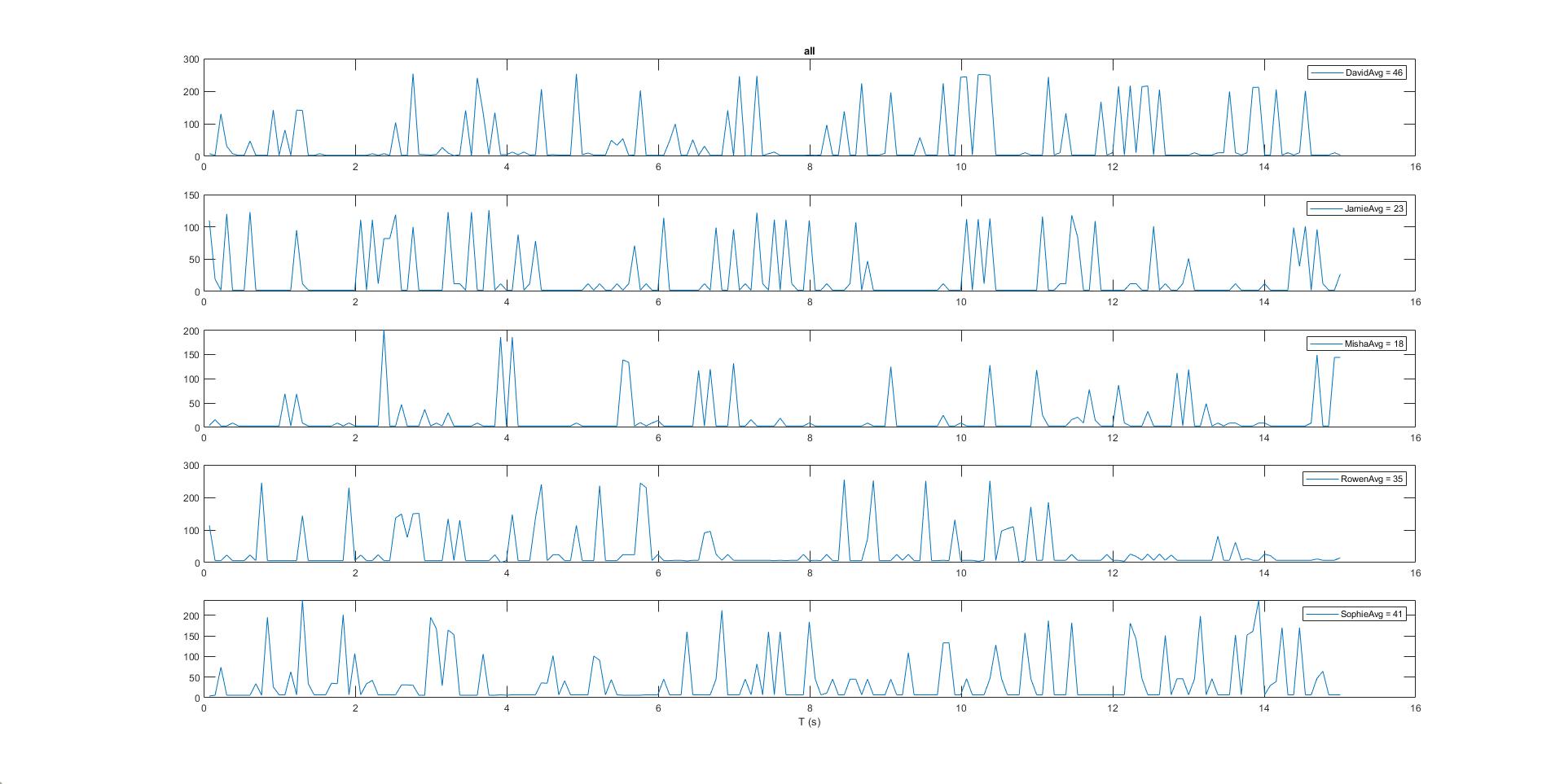

Supplement: S1 Appendix — (DOCX) [file pone.0285866.s009.docx]
